# Involvement of FKBP6 in hepatitis C virus replication

**DOI:** 10.1038/srep16699

**Published:** 2015-11-16

**Authors:** Hirotake Kasai, Kunihiro Kawakami, Hiromasa Yokoe, Kentaro Yoshimura, Masanori Matsuda, Jun Yasumoto, Shinya Maekawa, Atsuya Yamashita, Tomohisa Tanaka, Masanori Ikeda, Nobuyuki Kato, Toru Okamoto, Yoshiharu Matsuura, Naoya Sakamoto, Nobuyuki Enomoto, Sen Takeda, Hideki Fujii, Masayoshi Tsubuki, Masami Kusunoki, Kohji Moriishi

**Affiliations:** 1 Department of Microbiology, Faculty of Medicine, University of Yamanashi, Chuo-shi, Yamanashi 409-3898, Japan; 2 Faculty of Life and Environmental Sciences, University of Yamanashi, Kofu-shi, Yamanashi 400-8510, Japan; 3 Institute of Medical Chemistry, Hoshi University, 2-4-41 Ebara, Shinagawa-ku, Tokyo 142-8501, Japan; 4 Department of Anatomy and Cell Biology, Division of Medicine, Interdisciplinary Graduate School of Medicine and Engineering, University of Yamanashi, Chuo-shi, Yamanashi 409-3898, Japan; 5 Department of First Surgery, Faculty of Medicine, University of Yamanashi, Chuo-shi, Yamanashi 409-3898, Japan; 6 First Department of Internal Medicine, Faculty of Medicine, University of Yamanashi, Chuo-shi, Yamanashi 409-3898, Japan; 7 Department of Tumor Virology, Graduate School of Medicine, Dentistry, and Pharmaceutical Sciences, Okayama University, Okayama, Okayama 700-8530, Japan; 8 Department of Molecular Virology, Research Institute for Microbial Diseases, Osaka University, Suita, Osaka 565-0871, Japan; 9 Department of Gastroenterology and Hepatology, Hokkaido University Graduate School of Medicine, Sapporo, Hokkaido 060-8638, Japan

## Abstract

The chaperone system is known to be exploited by viruses for their replication. In the present study, we identified the cochaperone FKBP6 as a host factor required for hepatitis C virus (HCV) replication. FKBP6 is a peptidyl prolyl *cis-trans* isomerase with three domains of the tetratricopeptide repeat (TPR), but lacks FK-506 binding ability. FKBP6 interacted with HCV nonstructural protein 5A (NS5A) and also formed a complex with FKBP6 itself or FKBP8, which is known to be critical for HCV replication. The Val^121^ of NS5A and TPR domains of FKBP6 were responsible for the interaction between NS5A and FKBP6. FKBP6 was colocalized with NS5A, FKBP8, and double-stranded RNA in HCV-infected cells. HCV replication was completely suppressed in FKBP6-knockout hepatoma cell lines, while the expression of FKBP6 restored HCV replication in FKBP6-knockout cells. A treatment with the FKBP8 inhibitor *N*-(*N*′, *N*′-dimethylcarboxamidomethyl)cycloheximide impaired the formation of a homo- or hetero-complex consisting of FKBP6 and/or FKBP8, and suppressed HCV replication. HCV infection promoted the expression of FKBP6, but not that of FKBP8, in cultured cells and human liver tissue. These results indicate that FKBP6 is an HCV-induced host factor that supports viral replication in cooperation with NS5A.

Hepatitis C virus (HCV) affects 170 million individuals worldwide, and leads to liver disorders such as liver cirrhosis and hepatocellular carcinoma^1^. HCV is an enveloped RNA virus classified into the genus *Hepacivirus* of the family *Flaviviridae*. A single polyprotein of HCV is encoded by the positive-strand viral RNA genome with a nucleotide length of 9.6 kb, and is cleaved by viral and host proteases into 10 viral proteins. Nonstructural proteins, including p7, NS2, NS3, NS4A, NS4B, NS5A, and NS5B, play important roles in viral genomic replication and assembly, while structural proteins, including Core, E1, and E2, form the viral particles^2^. HCV infection alters inner membrane compartments, resulting in the formation of a convoluted membrane structure (called the membranous web), in which the viral genome is replicated by a replication complex consisting of viral and host factors^3^. NS5A is a membrane-anchored phosphoprotein characterized as a proline-rich hydrophilic and zinc-binding protein that regulates viral genomic RNA replication and viral particle assembly^4^. The full biological and virological functions of NS5A are not yet known.

Several viruses exploit the functions of the host chaperone machinery for their propagation and related pathogenesis. The viral oncoprotein Tax of human T-cell leukemia virus type 1 associates with the host Hsp90-chaperone system in order to avoid proteasomal degradation^5^. Hsp90 also ensures the correct folding of the conserved herpesvirus kinases at the lytic stage in order to improve the viral production of human cytomegalovirus or Epstein-Barr virus^6^. Previous findings suggest that Hsp90 directly and indirectly support HCV replication (refer to the review of Geller *et al.*^7^). Hsp90, which is highly conserved and ubiquitously expressed, contributes to conformational changes to endogenous proteins, including steroid receptors and cell signaling kinases^8^,^9^. Hsp90 hydrolyzes ATP into ADP and phosphoric acid, and markedly changes the conformation of a client protein^10^. On the basis of the chaperone cycle for steroid hormone receptors, the Hsp90 system consists of Hsp70/Hsp40 and several cochaperones, such as Hop/Sti, peptidyl prolyl *cis-trans* isomerases (PPIases), including three tetratricopeptide repeat (TPR) domains, and CDC37, among others^11^. The PPIase including TPRs interacts with the MEEVD motif of Hsp90 through its own two-carboxyl clamp position residues of TPR. Several members of the FK-506 binding protein (FKBP) family have TPR domains and exhibit the ability to bind Hsp90 through interactions between the MEEVD motif and TPR. Several cochaperones have been shown to provide the determinant of client proteins for the Hsp90 system.

Our earlier studies indicate that FKBP8/FKBP38 plays an important role in HCV replication, cooperating with other cochaperones^8^,^12^,^13^. FKBP8 is a member of the FKBP family and has a domain for FK-506 binding and PPIase. However, FKBP8 exhibits no ability to bind FK-506 because it lacks an essential residue^14^. FKBP8 has the ability to bind to NS5A and Hsp90 through its own TPR domains and is colocalized with NS5A, double-stranded RNA (dsRNA), Hsp90, and other cochaperones such as FKBP8 within the convoluted membrane structure of infected cells^12^. FKBP8 may provide a client determinant for NS5A in order to maintain efficient viral replication. FKBP52 (FKBP4), FKBP51 (FKBP5), FKBP36 (FKBP6), and FKBP8 have been classified into the TPR group of the FKBP family, which contains three tandem repeats of TPR^15^. In the present study, we determined involvement of FKBP4, FKBP5, and FKBP6 in NS5A binding and HCV replication.

## Results

### Identification of FKBP6 as an NS5A-binding host factor

**Figure 1:**
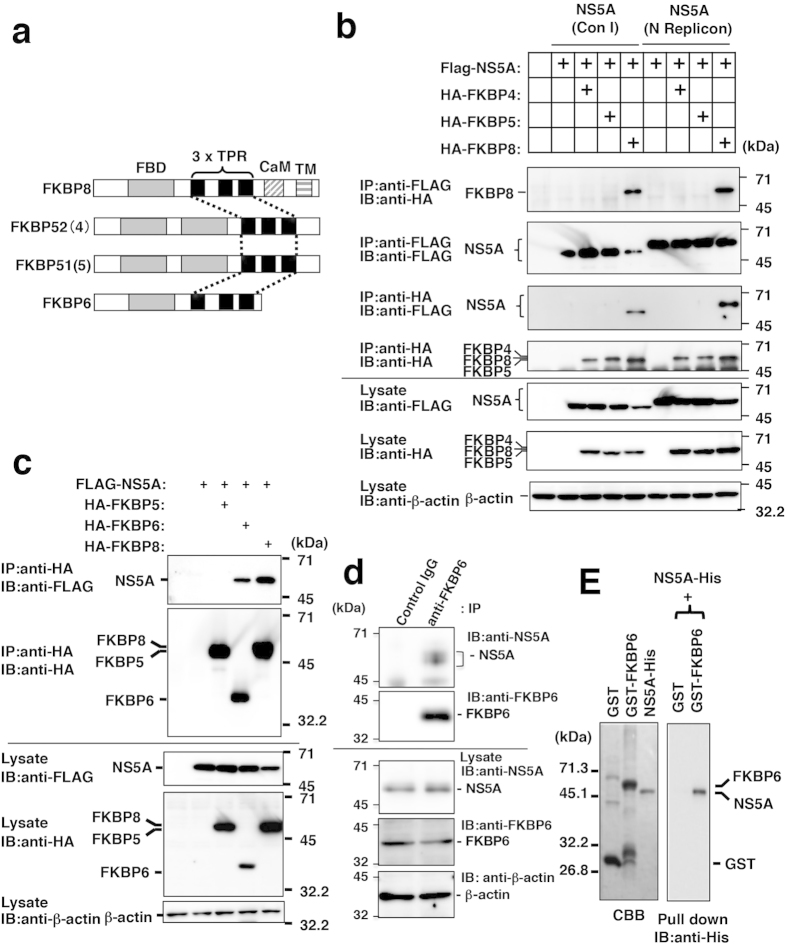
Interaction between FKBP6 and NS5A. (**a**) Schematic structures of FKBPs including three tandem repeats of the TPR domain are shown. (**b**) The region encoding NS5A was amplified from the total cDNA of strain Con1 (left half) or the N replicon cell line (right half). FLAG-NS5A derived from the Con1 (Control) or N strain was expressed with HA-FKBP4, −5, and −8 in 293T cells. Proteins in lysates were immunoprecipitated with an antibody to FLAG or HA epitope tag. Precipitated proteins were subjected to Western blotting. (**c**) FLAG-NS5A (Con1) was expressed with HA-tagged FKBP5, FKBP6, or FKBP8 in 293T cells. The transfected cells were harvested 48 h post-transfection and then lysed for immunoprecipitation. (**d)** The replicon cell line derived from the genotype 1b Con1 strain (clone 9-13) was lysed with the lysis buffer. The lysate was subjected to immunoprecipitation using goat anti-FKBP6 IgG and normal goat IgG. (**E**) Purified recombinant GST-FKBP6 and NS5A-His (Con1) were subjected to SDS-PAGE and stained with Coomassie brilliant blue R-250 (left panel). Both proteins were subjected to the GST-pulldown assay and Western blotting using an anti-6x His antibody. The data shown are representative of three independent experiments. Original blots in B, C, and D are shown in Supplementary Figures 1, 2, and 3, respectively.

Our previous findings suggest that the TPR domain of FKBP8 interacts with NS5A domain I in order to support HCV replication^8^,^12^. However, we did not investigate interactions of other members of the TPR group with NS5A. FKBP4, FKBP5, and FKBP6 may be functional molecules equivalent to FKBP8 because they possess three tandem repeats of the TPR domain, similar to FKBP8 (Fig. 1a). FLAG-tagged NS5A (FLAG-NS5A) was co-expressed with HA-tagged FKBP4 (HA-FKBP4), FKBP5 (HA-FKBP5), or FKBP8 (HA-FKBP8) in 293T cells and was subjected to immunoprecipitation (Fig. 1b). FLAG-NS5A (Con I or N strain) was immunoprecipitated with HA-FKBP8 using an anti-FLAG antibody, and HA-FKBP8 was also precipitated with FLAG-NS5A using an anti-HA antibody; however, the binding of NS5A with FKBP4 or FKBP5 was not detected (Fig. 1b). Thus, we examined the binding of FKBP6 to NS5A. HA-tagged FKBP6 (HA-FKBP6) and HA-FKBP8 (positive control), but not HA-FKBP5 (negative control), were precipitated with FLAG-NS5A using an anti-HA antibody (Fig. 1c). Endogenous FKBP6 was co-precipitated with functional NS5A in the replicon cell line (Fig. 1d). We investigated a direct interaction between NS5A and FKBP6. Recombinant C-terminally His_x6_-tagged NS5A (NS5A-His) and N-terminally glutathione S transferase (GST)-tagged FKBP6 (GST-FKBP6) were prepared in *E. coli*, and were purified using affinity resins (Fig. 1E, left panel). GST-FKBP6 and GST were each mixed with the NS5A-His protein at a molecular ratio of 1:1 and pulled down with glutathione-conjugated Sepharose 4B beads. The precipitated beads were then subjected to immunoblotting with an anti-His_x6_ antibody after SDS-PAGE. We detected precipitated NS5A-His by immunoblotting instead of protein staining in light at molecular sizes similar to those of GST-FKBP6 and NS5A-His. As shown in Fig. 1E (right panel), NS5A-His was co-precipitated with GST-FKBP6, but not with GST. These results suggest that FKBP6 directly binds HCV NS5A.

### A role of FKBP6 in HCV replication

**Figure 2:**
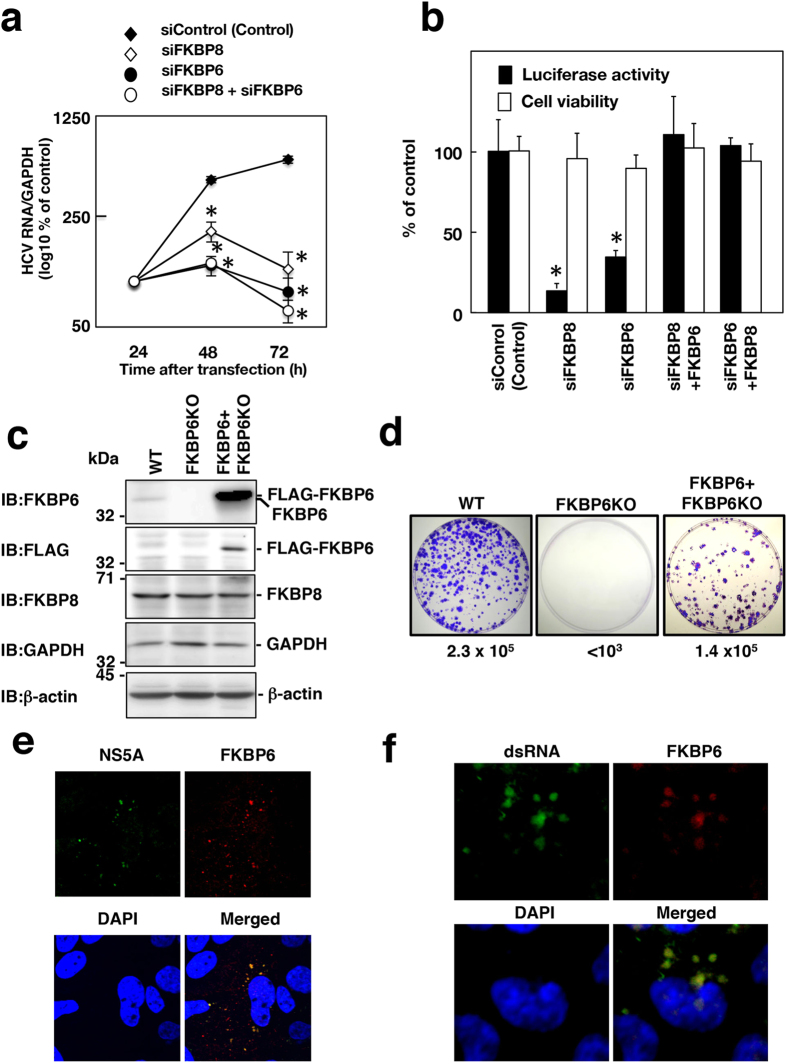
Effects of FKBP6 knockdown on HCV replication. (**a**) Huh7OK1 cells infected with HCVcc (JFH-1) were transfected with siFKBP8 and/or siFKBP6 at 10 nM. Culture supernatants were harvested from HCVcc-infected cells at the indicated times after transfection with 10 nM siRNAs. Viral RNA was estimated by qRT-PCR, normalized with the value of GAPDH mRNA and is presented as a percent of the control. (**b**) Replicon O cells were transfected with siFKBP6 or siFKBP8 at a final concentration of 10 nM and incubated for 16 h. The resulting cells were transfected again with an empty plasmid or a plasmid encoding FKBP8 or FKBP6, further incubated for 56 h and then harvested in order to measure luciferase activity and cell viability. The values obtained were standardized to those of the control cells. The amount of total transfected siRNA was adjusted with siControl in the absence of one of the siRNAs targeting FKBPs. Asterisks indicate a significant difference versus the control value (*P < 0.05). The data shown in this figure are representative of three independent experiments. (**c**) Cell lysates were prepared from Huh7OK1 cells (WT), FKBP6KO cells and FKBP6KO Huh7OK1 cells expressing FLAG-FKBP6 (FKBP6 + FKBP6KO), and were subjected to immunoblotting. (**d**) A colony formation assay was performed. Colony formation efficiency (colony number per μg of transfected subgenomic RNA) is shown under each panel. (**e**) Endogenous FKBP6 and NS5A in the infected cells were stained with the first antibodies and the secondary fluorescent antibodies, and observed under a confocal microscope at 400 times magnification. (**f**) Endogenous FKBP6 and double-stranded RNA (dsRNA) in the infected cells were stained with a goat anti-FKBP6 antibody and mouse anti-dsRNA antibody, followed by staining with an AF594-conjugated donkey antibody to goat IgG and an AF488-conjugated goat antibody to mouse IgG, respectively. The secondary antibody to goat IgG was reacted first in order to avoid a cross-reaction. The localization of the stained proteins was analyzed by confocal laser scanning microscopy. The stained samples were observed under a confocal microscope at 600 times magnification.

We examined the effects of FKBP6 on HCV infection. siRNA targeting FKBP6 (siFKBP6) or FKBP8 (siFKBP8) was transfected to Huh7 OK1 infected with HCVcc derived from the genotype 2a JFH1 strain. Culture supernatants of the transfected cells were harvested 24, 48, and 72 h post-transfection. Viral RNA in the culture supernatants was estimated by qRT-PCR. Viral production was impaired in JFH-1-infected Huh7OK1 by transfection with siFKBP6, siFKBP8, or both (Fig. 2a, and Supplementary Figure 9). The knockdown of FKBP6 also impaired replication of the subgenomic replicon at a level similar to the knockdown of FKBP8 (Fig. 2b). Knockout of the FKBP6 gene in the Huh7OK1 cell line was achieved using the CRISPR/Cas9 system for the colony formation assay. The deleted region in each allele was determined by direct sequencing as described in the Materials and Methods section. FKBP6 was weakly expressed in Huh7OK1 cells, but not in FKBP-knockout (FKBP6KO) Huh7OK1 cells, while FKBP8 was expressed at a similar level in Huh7OK1 and FKBP6KO cells (Fig. 2c). FLAG-FKBP6 was highly expressed in FKBP6KO Huh7OK1 cells expressing FLAG-FKBP6, but did not affect the expression of FKBP8 (Fig. 2c). The synthesized replicon RNA, carrying a neomycin resistance gene, was introduced into Huh-7OK1, FKBP6KO, or FLAG-FKBP6-expressing FKBP6KO cells. The resulting transfected cells were cultivated in the presence of 0.4 mg/ml G418 for 4 weeks. The remaining cell colonies were fixed and stained with crystal violet. The replicon RNA yielded 2.5 × 10^5^ colonies per μg of the transfected RNA in the Huh7OK1 cell line, but no colony in the FKBP6KO cell line (Fig. 2d). Furthermore, the colony formation efficiency of FKBP6KO cells expressing FLAG-FKBP6 was 1.4 × 10^5^ colonies per μg of the transfected RNA (Fig. 2d). Huh7 OK1 and FKBPKO Huh7OK1 cells were infected with HCVcc and then harvested at 4 days post-infections. Intracellular HCV RNA was 100 times decreased by the knockout of FKBP6 gene (Supplementary Figure 10). Endogenous FKBP6 was mainly localized in the cytoplasm as dot-like structures and co-localized with NS5A in Huh7 OK1 cells infected with HCVcc (Fig. 2e). FKBP6 was also co-localized with double-stranded RNA (dsRNA), the detection of which is a hallmark of viral replication, in the infected cells as dot-like structures (Fig. 2f). These findings suggest that FKBP6 is essential for HCV replication.

### Involvements of Val^121^ of NS5A and TPR domains of FKBP6 in the interaction

**Figure 3:**
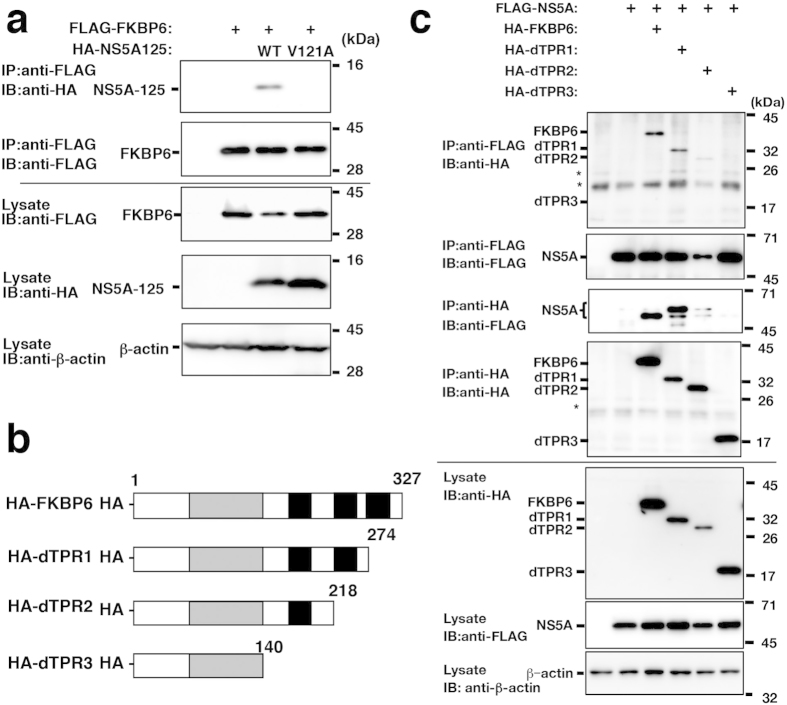
The TPR domain of FKBP6 and Val121 of NS5A are critical for their interaction. (**a**) The region spanning from the 1st to 125th positions of NS5A (HA-NS5A125wt) or a mutant in which Val was replaced with Ala at the 121st position (HA-NS5A125V121A) was expressed with FLAG-FKBP6 and then subjected to immunoprecipitation. Original blots are shown in Supplementary Figure 4. (**b**) Schematic diagram of the C-terminal deletion mutants of FKBP6 used in (c). (**c**) HA-tagged C-terminal deletion mutants of FKBP6 were expressed with FLAG-NS5A in 293T cells. The transfected cells were subjected to immunoprecipitation. Original blots are shown in Supplementary Figure 5. Asterisks indicate nonspecific bands. The results are representative of three independent assays.

Our previous findings suggested that domain I of NS5A (1–213) interacts with FKBP8^8,12^. Further mutational analyses of NS5A in our previous study suggest that the Val^121^ of NS5A is critical for the binding of NS5A’s N-terminal portion to FKBP8 and for viral replication^12^. We then investigated the role of Val^121^ of NS5A in binding to FKBP6. FLAG-FKBP6 was expressed with an N-terminally HA-tagged NS5A region spanning from amino acid residue 1 to 125 (HA-NS5A125wt) or with HA-NS5A125, the Val of which was replaced with Ala at the 121st position (HA-NS5AV121A). FLAG-FKBP6 was precipitated with HA-NS5A125wt, but not HA-NS5AV121A using an anti-FLAG antibody (Fig. 3a). Our previous findings suggest that the TPR domains of FKBP8 are required for the interaction between FKBP8 and NS5A^8^. C-terminal deletion mutants of FKBP6 were used in the present study (Fig. 3b) as follows: HA-dTPR1 was deficient in the C-terminal last TPR domain, HA-dTPR2 lacked the last two C-terminal TPR domains, and HA-TPR3 lacked all TPR domains. FLAG-NS5A was precipitated with wild-type FKBP6, dTPR1, and dTPR2 using an anti-FLAG antibody (Fig. 3c), while HA-dTPR2 was co-precipitated at a lower level than the wild type and HA-dTPR1 (Fig. 3c). In the reverse experiment, HA-FKBP6 wild type, HA-dTPR1, and HA-dTPR2 were each precipitated with FLAG-NS5A using an anti-HA antibody. HA-dTPR1 mainly precipitated FLAG-NS5A with a greater molecular weight than FLAG-NS5A precipitated with HA-FKBP6 wild-type, while FLAG-NS5A was precipitated with HA-dTPR2 at a lower level than with the wild type and dTPR1 (Fig. 3c). FKBP6 may regulate the phosphorylation state of NS5A in cooperation with Hsp90. FLAG-NS5A was not precipitated with HA-dTPR3 using an anti-FLAG antibody, and the interaction between FLAG-NS5A and HA-dTPR3 was not found in the reverse immunoprecipitation test (Fig. 3c). Furthermore, the overexpression of HA-FKBP6, but not that of HA-dTPR3, partially recovered HCV replication in FKBP6-knockdown replicon cells (Supplementary Figure 11). These results suggest that the Val^121^ of NS5A and complete TPR domains of FKBP6 are required for the full interaction between NS5A and FKBP6.

### A complex formation of FKBP6 with FKBP8 and/or FKBP6

**Figure 4:**
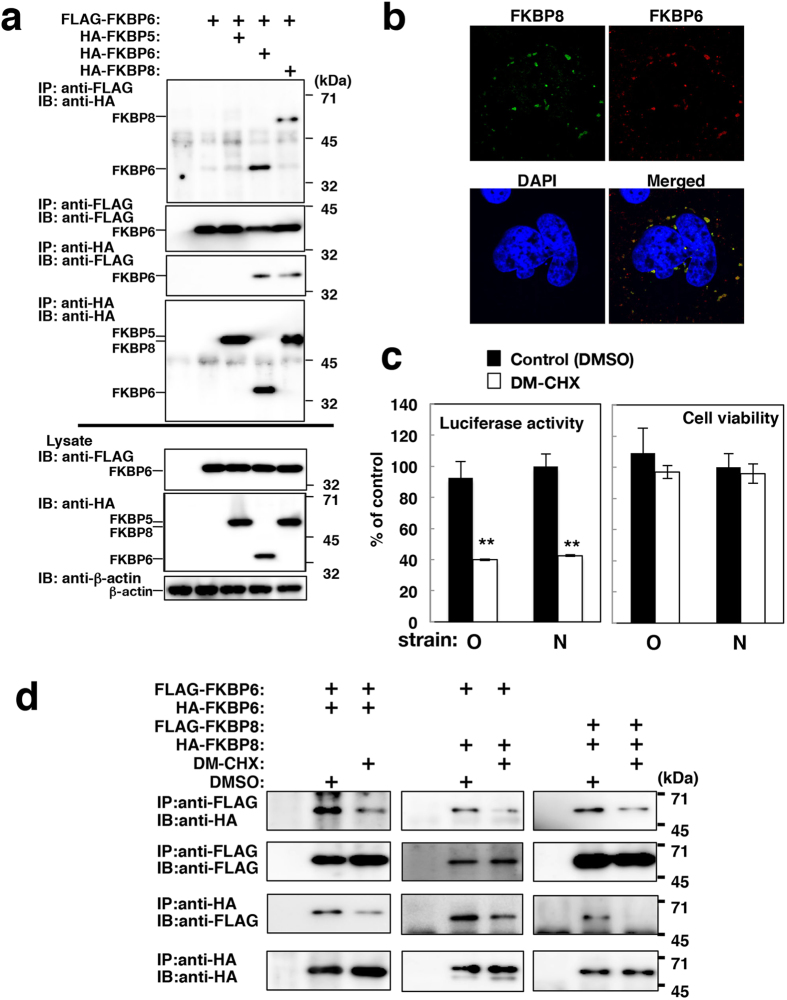
Multimerization of FKBP6 and FKBP8. (**a**) FLAG-FKBP6 was expressed with HA-tagged FKBP5, −6, and −8 in 293T cells. Cell lysates were prepared 48 h post-transfection and then subjected to immunoprecipitation. Original blots are shown in Supplementary Figure 6. (**b**) The intracellular localization of endogenous FKBP6 and FKBP8 was observed in JFH1-infected cells. The stained samples were observed under a confocal microscope at 600 times magnification. (**c**) Replicon cell lines derived from O and N strains (genotype 1b) were incubated with DMSO or DM-CHX (80 μM) for 24 h. Luciferase activity and cell viability were estimated 48 h after removing DMSO or DM-CHX and are presented as a percent of the control, which was the value of the N replicon cells treated with DMSO. Asterisks indicate a significant difference from the control value (**P < 0.01). (**d**) FLAG-FKBP6 or -FKBP8 was expressed with HA-FKBP6 or HA-FKBP8. The transfected cells were harvested 48 h post-transfection and lysed with lysis buffer. Half of the cell lysate was mixed with DMSO, while the other half was mixed with DM-CHX (80 μM). These mixtures were subjected to an immunoprecipitation test using an anti-FLAG or -HA antibody. The precipitates were subjected to Western blotting. Original blots are shown in Supplementary Figure 7. The data shown in this figure are representative of three independent experiments.

Our previous findings suggest a novel function for FKBP8, namely, it acts in multimerization^8^. In the present study, we examined a hetero- or homo-multimerization of FKBP6. FLAG-FKBP6 was expressed with HA-FKBP5, HA-FKBP6, or HA-FKBP8 in 293T cells. The resulting lysate was subjected to immunoprecipitation using an anti-FLAG or anti-HA antibody. FLAG-FKBP6 co-precipitated HA-FKBP6 and HA-FKBP8, but not HA-FKBP5 (Fig. 4a). In addition, the reverse experiment showed that HA-FKBP6 and HA-FKBP8 both co-precipitated FLAG-FKBP6, whereas HA-FKBP5 did not (Fig. 4a). Endogenous FKBP6 was co-localized with endogenous FKBP8 as dot-like structures in infected cells (Fig. 4b). Our results suggest that FKBP6 forms a complex with FKBP8 and/or FKBP6.

Edlich *et al.* reported that *N*-(*N*′, *N*′-dimethylcarboxamidomethyl)cycloheximide (DM-CHX) specifically inhibited the PPIase activity of FKBP8, but not that of other FKBP^16^. Although cell viability was not affected by the treatment with DM-CHX in the present study, HCV replication was significantly impaired in two replicon cell lines by the treatment with DM-CHX (Fig. 4c). We observed homo-multimerization involving FKBP6 or FKBP8 in the presence of DMSO, and the DM-CHX treatment impaired this homo-multimerization (Fig. 4d, left and right panels). Moreover, hetero-multimerization involving FKBP6 and FKBP8 was also impaired by the DM-CHX treatment (Fig. 4d, middle panels). These results suggest that the treatment with DM-CHX impairs HCV replication and disrupts the formation of complexes consisting of FKBP6 and/or FKBP8.

### Similar functions of FKBP6 and FKBP8 in HCV replication

**Figure 5:**
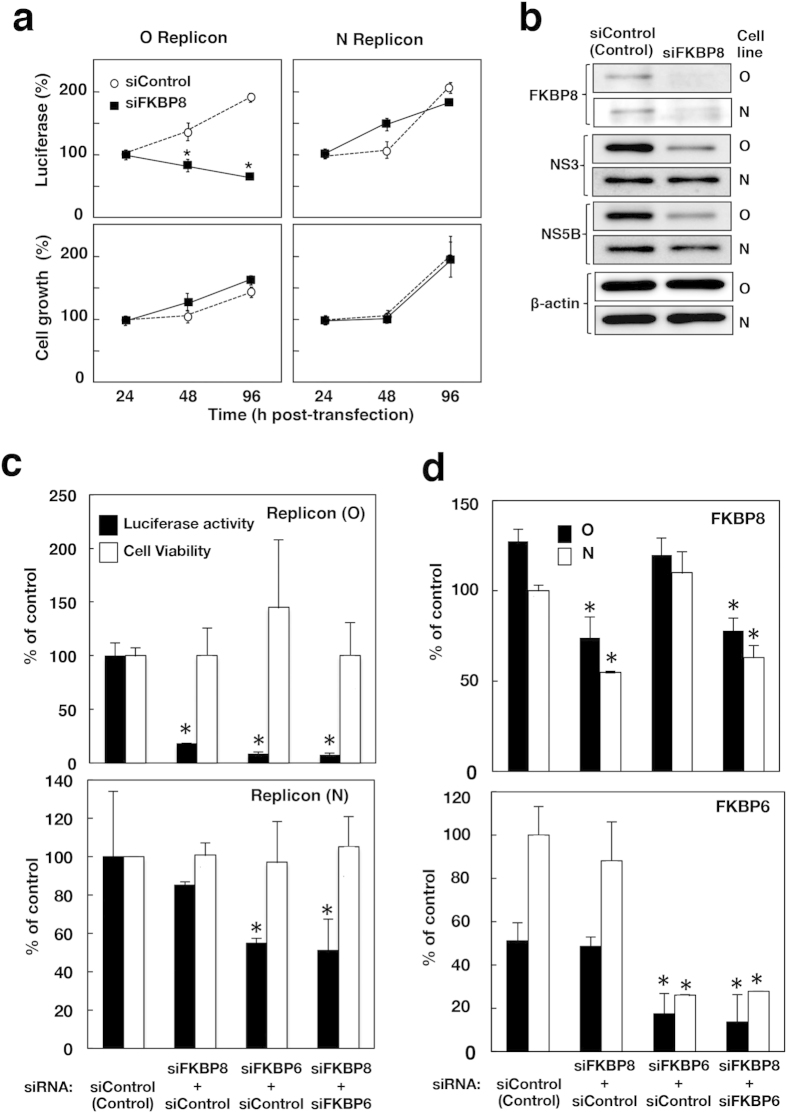
Effects of FKBP8 knockdown on the replication of replicons derived from O and N strains. (**a**) HCV replicon cell lines were transfected with siFKBP8 (closed squares, solid lines) or non-targeted siRNA siControl (open circles, broken lines) at a final concentration of 10 nM. Transfected cells were incubated in medium lacking G418 and then collected at the indicated time. Luciferase activity was evaluated and then normalized with the cell number (upper panels). Cell viability was measured by the method described in the Materials and Methods section (lower panels). Asterisks indicate a significant difference from the control value (*P < 0.05). (**b**) Cell lysates prepared from cells harvested 72 h post-transfection were then subjected to Western blotting. Original blots are shown in Supplementary Figure 8. (**c**) The cells were transfected with siRNAs at 10 nM each and then incubated for 72 h. Luciferase activity and cell viability were measured in the O replicon cell line (upper) and N replicon cell line (lower) transfected with siFKBP6 and/or siFKBP8. The values obtained were standardized with that of the control and are presented as a percentage of the control (siControl). (**d**) The transcriptional levels of FKBP8 (upper) and FKBP6 (lower) were measured by qRT-PCR. The values of FKBP mRNAs were normalized with that of GAPDH mRNA and are presented as a percent of the control of the N replicon cells. Asterisks indicate a significant difference from the control value (*P < 0.05).

The siRNA targeting FKBP8 (siFKBP8) was transfected into two subgenomic replicon cell lines derived from the O and N strains, which had not been examined for the influence of the knockdown of FKBP8. The transfected replicon cell lines were harvested 24 h, 48 h, and 96 h post-transfection. Since both replicon RNAs encode a chimeric protein consisting of luciferase and neomycin phosphotransferase^17^,^18^, luciferase activity corresponds to HCV replication. Luciferase activity decreased in a time-dependent manner in the cell line harboring subgenomic replicon RNA derived from strain O, designated the O replicon cell line in this study (Fig. 5a, upper left). However, luciferase activity was not impaired by the knockdown of FKBP8 in the cell line harboring subgenomic replicon RNA derived from strain N, designated as the N replicon cell line (Fig. 5a, upper right). The knockdown of FKBP8 did not alter the cell viability of either cell line from that of cells transfected with control siRNA (Fig. 5a, lower panels). Endogenous FKBP8 was reduced by the knockdown of FKBP8 in both cell lines (Fig. 5b). The knockdown of FKBP8 reduced the HCV proteins, NS3 and NS5B, in the O replicon cell line, but not in the N replicon cell line (Fig. 5b), suggesting that the down-regulation of luciferase activity is due to impaired viral replication. The viral RNA region encoding NS5A was amplified from the total cDNA of the N replicon cell line, and we determined the nucleotide sequence by direct sequencing. The K^20^, V^34^, N^272^, I^306^, V^375^, and S^405^ of NS5A deduced from the original database of the viral genome of strain N (GenBank accession No. AF139594) were replaced with R, A, T, V, A, and P, respectively, in NS5A amplified from the N replicon cell line. Val^121^ was conserved in NS5A proteins derived from the N replicon cell line as well as the original genome sequence. FLAG-NS5A derived from the N replicon cell line was immunoprecipitated with HA-FKBP8 using an anti-FLAG antibody, and HA-FKBP8 was also precipitated with FLAG-NS5A using an anti-HA antibody (Fig. 1b).

Single transfection with siFKBP8 strongly impaired HCV replication in the O replicon cell line (Fig. 5c, upper), but not in the N replicon cell line (Fig. 5c, lower), which is consistent with the results shown in Fig. 5a. On the other hand, single transfection with siRNA targeting FKBP6 (siFKBP6) impaired HCV replication in the O and N replicon cell lines (Fig. 5c, upper and lower). FKBP8 was expressed at similar levels in the two cell lines (Fig. 5d, upper), whereas FKBP6 was expressed at a higher level in the N replicon cell line than in the O replicon cell line (Fig. 5d, lower). The knockdown of FKBP8 reduced the expression of FKBP8 to a similar extent in the two cell lines, but did not affect the expression of FKBP6 or cell viability in either cell line (Fig. 5d, lower). The knockdown of FKBP6 reduced the amount of FKBP6 to a similar extent in the O and N replicon cell lines (Fig. 5d, lower), but did not affect the expression of FKBP8 or cell viability (Fig. 5c). Knockdown levels of FKBP6 and FKBP8 were also observed by western blotting analyses (Supplementary Figure 12). HCV replication was reduced by the knockdown of FKBP8 in the O replicon cell line, while the overexpression of FKBP6 restored HCV replication in the FKBP8-knockdown cell line (Fig. 2b). Furthermore, the knockdown of FKBP6 impaired HCV replication in the O replicon cell line (Fig. 2b), while the overexpression of FKBP8 restored HCV replication in the FKBP6-knockdown cell lines (Fig. 2b). These results suggest that FKBP6 and FKBP8 share similar functional characteristics in HCV replication.

### Induction of FKBP6 expression by HCV infection

**Figure 6:**
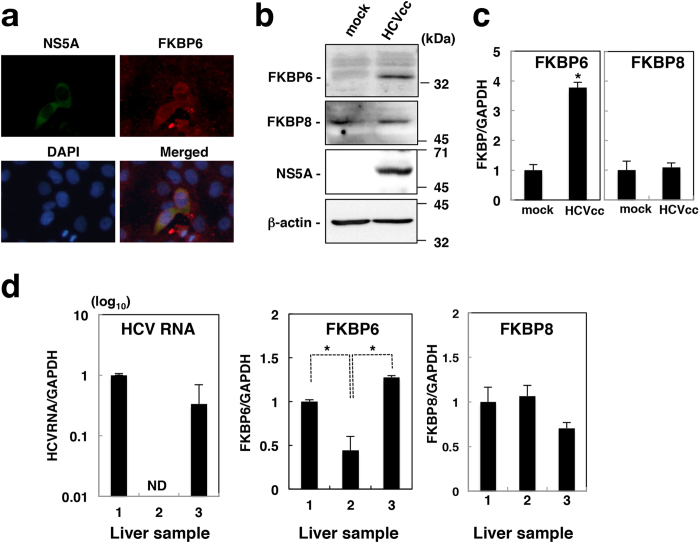
Effects of HCV replication on FKBP6 expression. (**a**) The stained cells used in Fig. 2e were observed at 200 times magnification using the fluorescence microscope BZ-9000 (Keyence, Osaka, Japan), which is not a confocal microscope. (**b**) Naïve and HCVcc-infected Huh7OK1 cells were harvested and then subjected to immunoblotting using antibodies to FKBP6, FKBP8, NS5A, and beta-actin. (**c**) FKBP6, FKBP8, and GAPDH mRNAs were estimated by qRT-PCR in naïve and HCVcc-infected cells, as described for the cells used in (**b**). The values obtained for FKBP6 and FKBP8 mRNAs were normalized with that of GAPDH mRNA and are presented as levels relative to the control (mock). Asterisks indicate a significant difference from the control value (*P < 0.05). (**d**) Human non-cancerous liver tissues (no. 1, 2, and 3) were obtained from three different liver samples of one donor. The HCV RNAs and mRNAs of FKBP6 and FKBP8 were estimated by qRT-PCR, normalized with GAPDH mRNA, and are presented as values relative to tissue no. 1. ND means “not detected”. The data shown in this figure are representative of three independent experiments.

Infected cells (NS5A-positive cells) were more potently stained with an anti-FKBP6 antibody than HCV-negative cells in the same field of view (Fig. 6a). Thus, we examined the effects of HCV infection on the expression of FKBP6 in cultured cells and the human livers. FKBP6 was more highly expressed at the protein and mRNA levels in HCVcc-infected cells than in non-infected cells (mock), whereas the expression of FKBP8 remained unchanged in HCV-infected cells and mock controls (Fig. 6a,c). Furthermore, a treatment with daclatasvir successfully remove HCV form HCV-infected cells and decreased the amount of FKBP6, corresponding to downregulation of HCV (Supplementary Figure 13). In human non-cancerous liver, FKBP6 was expressed at a significantly higher level in HCV-positive tissue than in HCV-negative tissue (Fig. 6d), whereas FKBP8 was not promoted more in HCV-positive tissue than in HCV-negative tissue (Fig. 6d). The data using additional liver samples also showed HCV-dependent upregulation of FKBP6 (Supplementary Figure 14). These results suggest that the expression of FKBP6 is promoted *ex vivo* and *in vivo* by HCV infection.

## Discussion

Hsp90 is composed of an N-terminal ATPase domain and C-terminal dimerization domain. It generally associates with other chaperone systems and cochaperones. The Hsp70 system partially folds a client protein, following recognition of the client protein by Hsp90. Hsp72, which has been classified into the Hsp70 family, positively regulates viral genomic replication, viral IRES activity, and the stability of the viral replication complex^19^. We previously reported that HCV NS5A also interacts with B-ind1 to maintain HCV replication through cochaperone activity, similar to p23^13^,^20^. Some of the client specificity of Hsp90 is determined by these collaborating cochaperones. CDC37 interacts with client cellular kinase and Hsp90 for the specific and correct folding of client kinases by Hsp90^21^. Our previous findings and the results of the present study suggest that FKBP6 and/or FKBP8 provide specificity to the chaperone system for HCV replication.

Knockout of the FKBP6 gene completely suppressed HCV replication on the basis of the colony formation assay (Fig. 2d), while the expression of FKBP6 restored HCV replication in FKBP6-knockout cells (Fig. 2d). Moreover, the knockdown of FKBP6 impaired HCV replication in infected cells and the O and N replicon cell lines irrespective of the expression level of FKBP8, suggesting that FKBP6 is a functional counterpart of FKBP8, but is more essential for the stable replication of HCV. FKBP6 was more highly expressed in the N replicon cell line than in the O replicon cell line (Fig. 5d), whereas FKBP8 was expressed at similar levels in both cell lines. The knockdown of FKBP6 or FKBP8 did not affect the expression of FKBP8 or FKBP6, respectively (Fig. 5c). The slight effects of the knockdown of FKBP8 on HCV replication in the N replicon may have been due to the high expression of FKBP6 in N replicon cells. In addition, there is other possibility that an unknown factor other than FKBP6/8 may support HCV replication instead of FKBP6/8 in the N replicon, because the knockdown of FKBP6 reduced HCV replication in N replicon cells to a lesser extent than in O replicon cell line (Fig. 5c). The mechanism of transcriptional regulation of FKBP6 has not been clarified yet. Several reports suggested that FKBP6 is expressed at lower levels or not detected in the liver under physiological conditions^22^,^23^. Our present results indicate that the expression of FKBP6 is accelerated more *ex vivo* and *in vivo* in infected cells than in naïve or cured cells (Fig. 6). FKBP8 may be involved in the early stage of HCV infection, whereas FKBP6 may persistently support HCV replication after viral entry.

NS5A has two kinds of phosphorylated states: p56 and p58. NS5A p56 and p58 represent the basal phosphorylated state and hyperphosphorylated state, respectively. Casein kinase II was previously reported to be involved in the basal phosphorylated state^24^,^25^, while casein kinase I alpha and Polo-like kinase I were reported to be responsible for the hyperphosphorylated state^26^,^27^. A recent study suggests that PI4K III alpha binds to the C-terminal region of NS5A domain I and upregulates the production of p56 and synthesis of phosphatidylinositol 4-phosphate^28^. Deletion of the last two C-terminal TRP domains of FKBP6 did not result in the complete loss of the ability to bind NS5A (Fig. 3c). However, NS5A precipitated by dTPR1 or dTPR2 had a slightly higher molecular weight than that by wild-type FKBP6. The last TPR domain includes two carboxylate clamp positions for Hsp90 binding (Fig. 3c). FKBP6 may affect the phosphorylation state of NS5A in cooperation with Hsp90. These results suggest that all three TPR domains of FKBP6 are required for the full ability of NS5A binding.

Jarczowski *et al.* reported that FKBP6 is an inhibitor of glyceraldehyde-3-phosphate dehydrogenase (GAPDH) in COS-7 because the expression of FKBP6 causes a significant reduction in GAPDH levels^29^. In the present study, knockout of the FKBP6 gene did not affect the expression of GAPDH in Huh7 OK1 cells (Fig. 2c). Loss of the FKBP6 gene in mice was previously shown to result in male infertility^22^, which is a hallmark of a deficiency in the PIWI-interacting RNA (piRNA) pathway^30^,^31^. Xiol *et al.* suggested that a chaperone complex consisting of FKBP6 and Hsp90 is required for the biogenesis of piRNA and silencing of retrotransposons^32^. The retrotransposon LINE1, which is a major source of endogenous mutagenesis, is silenced by the piRNA-dependent methylation of the LINE1 promoter^31^. The expression of LINE1 was previously found to be promoted in the human hepatocellular carcinoma of HBV- and HCV-positive patients by hypomethylation of the LINE1 promoter, thereby leading to the activation of an oncogenic pathway induced by the insertion of LINE1 upstream of tumor suppressor genes^33^. HCV replication may sequester FKBP6 in replication compartments and consume FKBP6 from the allocation for LINE1 inactivation, leading to the preservation of LINE1 activation. Further studies are needed in order to clarify the mechanisms underlying the involvement of FKBP6 in HCV replication and its pathogenesis.

## Materials and Methods

### Cell lines, virus, and reagents

The cell lines used in this study were maintained in Dulbecco’s modified Eagle’s medium (DMEM) containing 10% fetal calf serum (FCS) and 0.2 mg/mL G418. The Huh7/ORN3-5B #24 cell line, which harbors the subgenomic replicon RNA of the O strain (genotype 1b), was designated as the O replicon cell line in the present study^17^. The Huh7 Rep Feo cell line, which harbors the subgenomic replicon RNA of the N strain (genotype 1b)^18^, was designated as the N replicon cell line. The Huh7OK1 cell line, which is highly permissive to JFH-1 virus infection, was established as reported previously^12^. Viral RNA derived from the plasmid pJFH1 was transcribed and introduced into Huh7OK1 cells according to the method of Wakita *et al.*^34^. Huh7OK1 cells were infected with JFH1 at a multiplicity of infection of 0.1 and then passaged two times for 4 days each time. The resulting cells were used as the infected cells in the present study. The FKBP8 inhibitor *N*-(*N’*, *N’*-dimethylcarboxamidomethyl)cycloheximide (DM-CHX) was synthesized from cycloheximide by the method reported by Edlich *et al.*^16^. Subgenomic replicon RNA derived from genotype 1b strain Con1^35^ was transfected into Huh7OK1 cell lines. The transfected cells were incubated in the presence of 0.4 mg/ml G418. The remaining cells were used for the Con1 replicon cells based on Huh7OK1. The human embryonic kidney 293T cell line was purchased from the American Type Culture Collection (Manassas, VA, USA).

### Effects of the FKBP8 inhibitor DM-CHX on FKBP or FKBP-NS5A multimerization

We co-transfected the expression plasmid encoding FLAG-FKBP6, FLAG-FKBP8, or FLAG-NS5A with the expression vector of HA-FKBP6 or HA-FKBP8 into 293T cells on 6-well plates. The cells were lysed with 1% Nonidet P-40 lysis buffer consisting of 50 mM HEPES (pH 7.4), 1% Nonidet P-40, 150 mM NaCl, 0.1 mM EDTA, and 0.1% protease inhibitor cocktail (Calbiochem/EMD Millipore, San Diego, CA, USA). The lysate was immunoprecipitated using an anti-FLAG or anti-HA antibody with or without 80 μM DM-CHX. The precipitated proteins were subjected to 12.5% sodium dodecyl sulfate-polyacrylamide gel electrophoresis (SDS-PAGE) and Western blotting.

### Transfection, immunoblotting, immunoprecipitation, and gene silencing

Plasmid DNA was transfected into Huh7OK1 cells or 293T cells using Lipofectamine LTX with Plus reagent (Life Technologies, Carlsbad, CA, USA). Immunoprecipitation tests were performed as described previously^36^. Cell lysates were subjected to SDS-PAGE and then transferred onto polyvinylidene difluoride membranes (Merck Millipore, Billerica, MA, USA). Proteins transferred onto the membranes were reacted with an appropriate antibody, followed by a reaction with a horseradish peroxidase-conjugated antibody to rabbit or mouse IgG. The immunoreactive bands were immersed in Super Signal West Femto (Thermo Scientific, Rockford, IL, USA) and then visualized using an LAS4000mini imaging system (GE Healthcare, Uppsala, Sweden). The siRNAs targeted to FKBP6 or FKBP8 were synthesized by Life Technologies (Austin, TX, USA) and were introduced into the cell lines using Lipofectamine RNAiMax (Life Technologies). siRNAs with the Ambion® siRNA ID number s16085 were designated as siFKBP6. Control siRNA (siControl Non-targeting siRNA #2, Dharmacon®) was purchased from Thermo Scientific (Brebières, France), and siRNA targeting FKBP8 was as described by Shirane *et al.*^37^.

### Preparation of recombinant proteins and the GST pull-down method

The plasmid pET-UbCHis-del32-NS5A encoding the C-terminally His_x6_-tagged NS5A lacking the membrane -anchoring region (NS5A-His) and *Escherichia coli* strain BL21(DE3)pCG1 were kind gifts from Dr. C. E. Cameron^38^. The plasmid pGEX-FKBP6^29^, coding glutathione S-transferase-fused FKBP6 (GST-FKBP6), was kindly provided by Drs. Frank Edlich and Guntar Fisher. The protein purification procedures were as previously described^12^ and partially performed by the method of Huang *et al.*^38^. Briefly, *E. coli* strain BL21(DE3)pCG1 was transformed with pET-UbCHis-del-NS5A. BL21(DE3) was transformed with pGEX-FKBP6 or pGEX-4T1^29^. An overnight culture of each transformant was added at 1/50 volume into 250 ml of 2 ×YT medium. The resulting mixture was incubated at 37 °C, with shaking at 200 rpm. Isopropyl beta-thiogalactoside was added at a final concentration of 0.5 mM to the culture at 37 °C for 4 h, with shaking at 200 rpm. After centrifugation at 3000 × g for 5 min, the cell debris was washed once with PBS. The cell debris was suspended in 5 ml of 100 mM Tris-HCl (pH 8.0) containing 200 mM NaCl, and 10 mM 2-mercaptoethanol (2-ME), 0.5% Nonidet P-40, protease inhibitors (Roche, Mannheim, Germany), and 0.2 μg/ml lysozyme (Lysis buffer), incubated at 4 °C for 1 h, and then subjected to freezing/thawing once. The resulting mixture was sonicated at 4 °C for 5 min and then treated with 0.02 mg of DNase I per ml at room temperature for 5 min. This suspension was centrifuged at 10,000 × g at 4 °C for 30 min. The resulting supernatant of the transformant containing pET-UbCHis-del-NS5A was applied to 1 ml of nickel agarose (Sigma, St. Louis, MO, USA) at 4 °C. The nickel-charged resin was washed twice by spinning down with Lysis buffer containing 10 mM imidazole. The recombinant protein NS5A-His was eluted from the nickel resin with Lysis buffer containing 0.25 M imidazole and then dialyzed in PBS. GST-FKBP6 was eluted with 20 mM glutathione and then dialyzed in PBS. Protein concentrations were measured using the Bio-rad Protein Assay kit (BioRad, Hercules, CA, USA).

In the GST-pull down assay, purified His-NS5A and GST-FKBP6 (or GST) were mixed at an equal molar ratio and then rotated at 4 °C for 2 h. The resulting mixture was incubated with 25 μl of 50% glutathione-conjugated Sepharose 4B beads (GE Healthcare) for a further 30 min. The resulting beads were precipitated by centrifugation at 3000 × g for 5 min. The precipitated beads were washed three times with PBS. The resulting beads were suspended in 10 μl of SDS-PAGE loading buffer and subjected to SDS-PAGE. The gels were stained with Coomassie brilliant blue R-250 or subjected to Western blotting.

### Estimation of luciferase activity and cell viability

HCV replicon cells were seeded at 2 × 10^4^ cells per well in a 48-well plate 24 h before being treated. siRNAs targeting FKBP8 (siFKBP8), FKBP6 (siFKBP6), and/or control were transfected at a final concentration of 20 nM with Lipofectamine RNAi max reagent (Life Technologies). The treated cells were harvested at the indicated time after transfection and lysed in cell culture lysis reagent (Promega, Madison, WI, USA) or *Renilla* luciferase assay lysis buffer (Promega). Luciferase activity in the harvested cells was estimated with a luciferase assay system (Promega) or *Renilla* luciferase assay system (Promega). The resulting luminescence was detected using Luminescencer-JNR AB-2100 (ATTO, Tokyo, Japan) and corresponded to the expression level of the HCV replicon.

### Quantitative reverse-transcription polymerase chain reaction (qRT-PCR)

Total RNAs were prepared from cells and culture supernatants using an RNeasy Mini kit (Qiagen, Tokyo, Japan) and QIAamp Viral RNA Mini kit (Qiagen), respectively. First-strand cDNA was synthesized using a high-capacity cDNA reverse transcription kit (Life Technologies) with random primers. Each cDNA was estimated using a Fast SYBR Green master mix (Life Technologies) according to the manufacturer’s protocol. The fluorescent signals of SYBR Green were analyzed using ABI Step One Plus (Life Technologies). The HCV internal ribosomal entry site (IRES) region and human GAPDH gene were amplified using the respective primer pairs, as described previously^13^. The value of the HCV genome was normalized to that of GAPDH mRNA. Each PCR product was detected as a single band of the correct size upon agarose gel electrophoresis.

### Immunofluorescent staining

Huh7OK1 cells were infected with JFH1 HCVcc, passaged every four days, harvest at 12 days postinfection and then seeded at 0.5 × 10^4^ cells/well on glass cover slips. The cells were washed twice with phosphate-buffered saline (PBS) and then fixed with 4% paraformaldehyde at room temperature for 20 min. The cells were washed twice with PBS after fixation, permeabilized for 15 min at room temperature with PBS containing 0.3% saponin, and then incubated in PBS containing 3% bovine serum albumin (PBS-BSA) to block nonspecific reactions.

Nuclei were stained with 50 μM DAPI. These cells were incubated at 4 °C overnight in PBS-BSA containing a goat anti-FKBP6, mouse anti-FKBP8, and/or rabbit anti-NS5A antibody. Cells were washed three times with PBS-BSA and incubated at room temperature for 120 min in PBS-BSA containing appropriate Alexa Fluor (AF)-conjugated secondary antibodies (Molecular Probes®, Life Technologies, Eugene, OR). These cells were washed three times with PBS-BSA and then observed using a confocal microscope (FV1000, Olympus, Tokyo) or fluorescent microscope (BZ-9000, Keyence, Osaka, Japan).

### Gene knockout by the CRISPR/Cas9 system

FKBP6-knockout (FKBP6KO) Huh7OK1 cells were established following the CRISPR/Cas9 method reported by Fujihara *et al.*^39^. An FKBP6-targeting sequence composed of 5′- GGGGGAAGCGCGTTAAACCAGG -3′ was employed for the construction of plasmids encoding the guide RNA, pX330. The targeting DNA regions were amplified from the genomic DNA of Huh7OK1 cells using the primer pairs of 5′-CAACCACTGAGGATCCCGTCGGTAGGGGTCTGCC-3′ and 5′-TGCCGATATCGAATTCAAATCCACTTCAAACAAAGCATTTCCAA-3′, and were then introduced into pCAG EGxxFP. The pX330 and pCAG EGxxFP plasmids were both obtained from Addgene (plasmids #42230 and #50716; Cambridge, MA, USA). Huh7OK1 cells were transfected with these plasmids using Lipofectamine LTX. GFP-positive cells were isolated 48 h post-transfection using the cell sorter FACSAria^TM^ (BD Bioscience). Single cell clones were established by a colony isolation technique. The deleted regions of each allele of the FKBP6 gene were confirmed by direct sequencing. FKBP6 mRNA transcribed from each allele of the FKBPKO cell line was expected to lack the region of accagggagtcctggaaggggacgacgcccccggccagtccctgtacgagcggttaagtcagaggatgctg or taaaccagggagtcctggaaggggacgacgcccccggccag. The expression of FKBP6 was confirmed by an immunoblotting analysis. FKBP6KO cells were transfected with the plasmid encoding FLAG-FKBP6 and then incubated in the presence of puromycin. The surviving cells were isolated as a colony and then used in a colony formation assay. This colony formation assay was performed by a previously described method^35^,^40^. Huh7OK1 or FKBP-knockout cells were transfected with RNA synthesized on the basis of pFK-I_389_ neo/NS3-3′/NK5.1^35^ linearized by *Sca*I digestion.

### Statistical analysis

The measured values are shown as the mean ± standard deviation (S.D.). The significance of differences in the means was determined by the Student’s *t*-test.

### Ethics statement

Human peritumoral non-cancerous liver tissues were obtained from one patient infected with HCV who was followed up at Yamanashi University Hospital. The study protocol was approved by the Human Ethics Review Committee of Yamanashi University Hospital. This study followed the principles of the ethical guidelines of the Declaration of Helsinki. All participants provided written informed consent.

## Additional Information

**How to cite this article**: Kasai, H. *et al.* Involvement of FKBP6 in hepatitis C virus replication. *Sci. Rep.* **5**, 16699; doi: 10.1038/srep16699 (2015).

## Supplementary Material

Supplementary Information
