# Supplementary material for: Involvement of FKBP6 in hepatitis C virus replication
Source: Sci Rep. 2015 Nov 16;5:16699. doi: 10.1038/srep16699 (PMC4644952; doi:10.1038/srep16699)
Supplement: Supplementary Information [file srep16699-s1.pdf]

Supplementary Information

Involvement of FKBP6 in hepatitis C virus replication

Hirotake Kasai[1,*], Kunihiro Kawakami[2,*], Hiromasa Yokoe[3], Kentaro Yoshimura[4], Masanori Matsuda[5], Jun Yasumoto[1], Shinya Maekawa[6], Atsuya Yamashita[1], Tomohisa Tanaka[1], Masanori Ikeda[7], Nobuyuki Kato[7], Toru Okamoto[8], Yoshiharu Matsuura[8], Naoya Sakamoto[9], Nobuyuki Enomoto[6], Sen Takeda[4], Hideki Fujii[5], Masayoshi Tsubuki[3], Masami Kusunoki[2], and Kohji Moriishi[1]

[1]Department of Microbiology, Faculty of Medicine, University of Yamanashi, Chuo-shi, Yamanashi 409-3898, Japan
[2]Faculty of Life and Environmental Sciences, University of Yamanashi, Kofu-shi, Yamanashi 400-8510, Japan
[3]Institute of Medical Chemistry, Hoshi University, 2-4-41 Ebara, Shinagawa-ku, Tokyo 142-8501, Japan
[4]Department of Anatomy and Cell Biology, Division of Medicine, Interdisciplinary Graduate School of Medicine and Engineering, University of Yamanashi, Chuo-shi, Yamanashi 409-3898, Japan
[5]Department of First Surgery, Faculty of Medicine, University of Yamanashi, Chuo-shi, Yamanashi 409-3898, Japan
[6]First Department of Internal Medicine, Faculty of Medicine, University of Yamanashi, Chuo-shi, Yamanashi 409-3898, Japan
[7]Department of Tumor Virology, Graduate School of Medicine, Dentistry, and Pharmaceutical Sciences, Okayama University, Okayama, Okayama 700-8530, Japan
[8]Department of Molecular Virology, Research Institute for Microbial Diseases, Osaka University, Suita, Osaka 565-0871, Japan
[9]Department of Gastroenterology and Hepatology, Hokkaido University Graduate School of Medicine, Sapporo, Hokkaido 060-8638, Japan

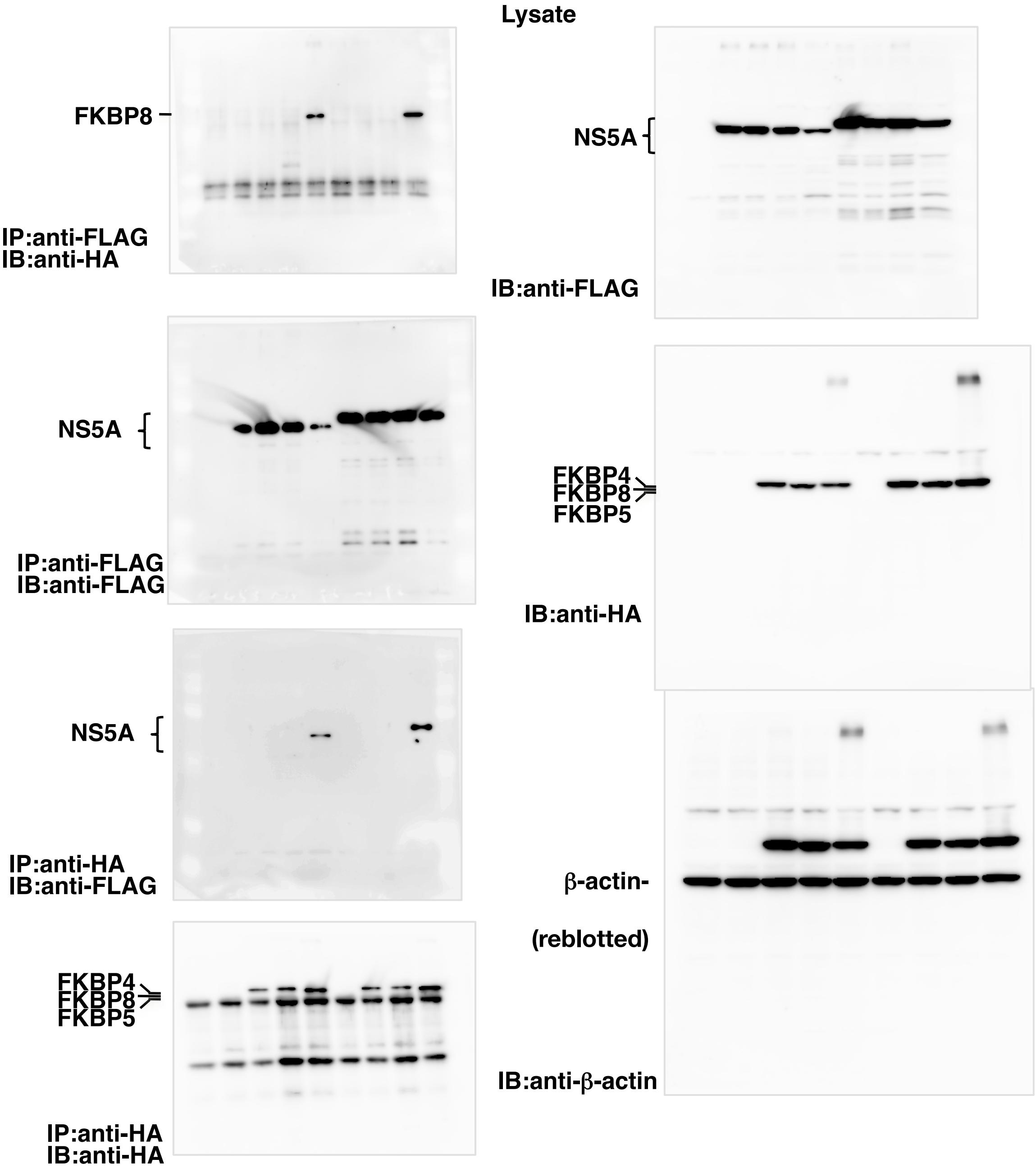

Supplementary Figure 1. Original data of Figure 1B.

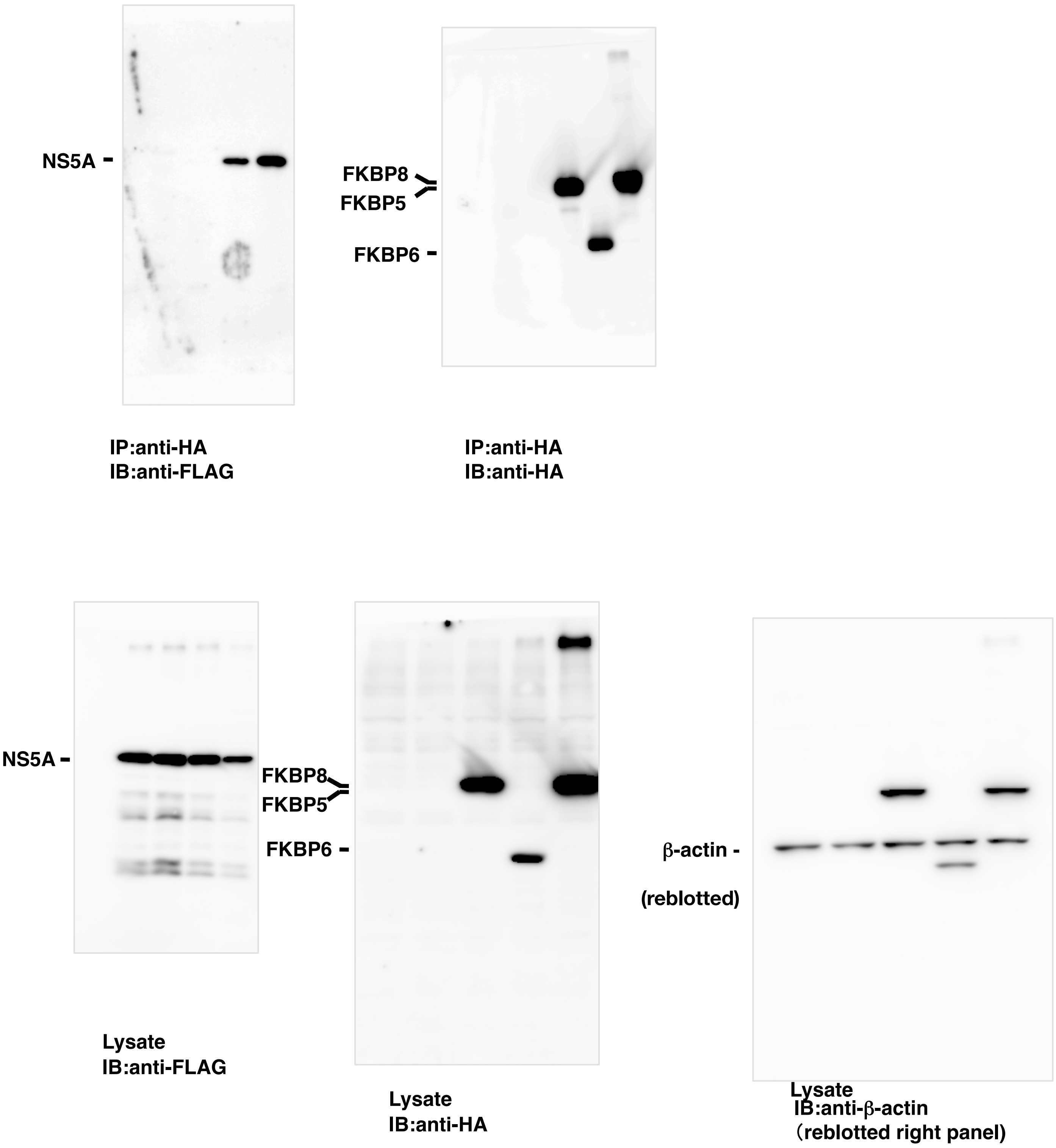

Supplementary Figure 2. Original data of Figure 1C.

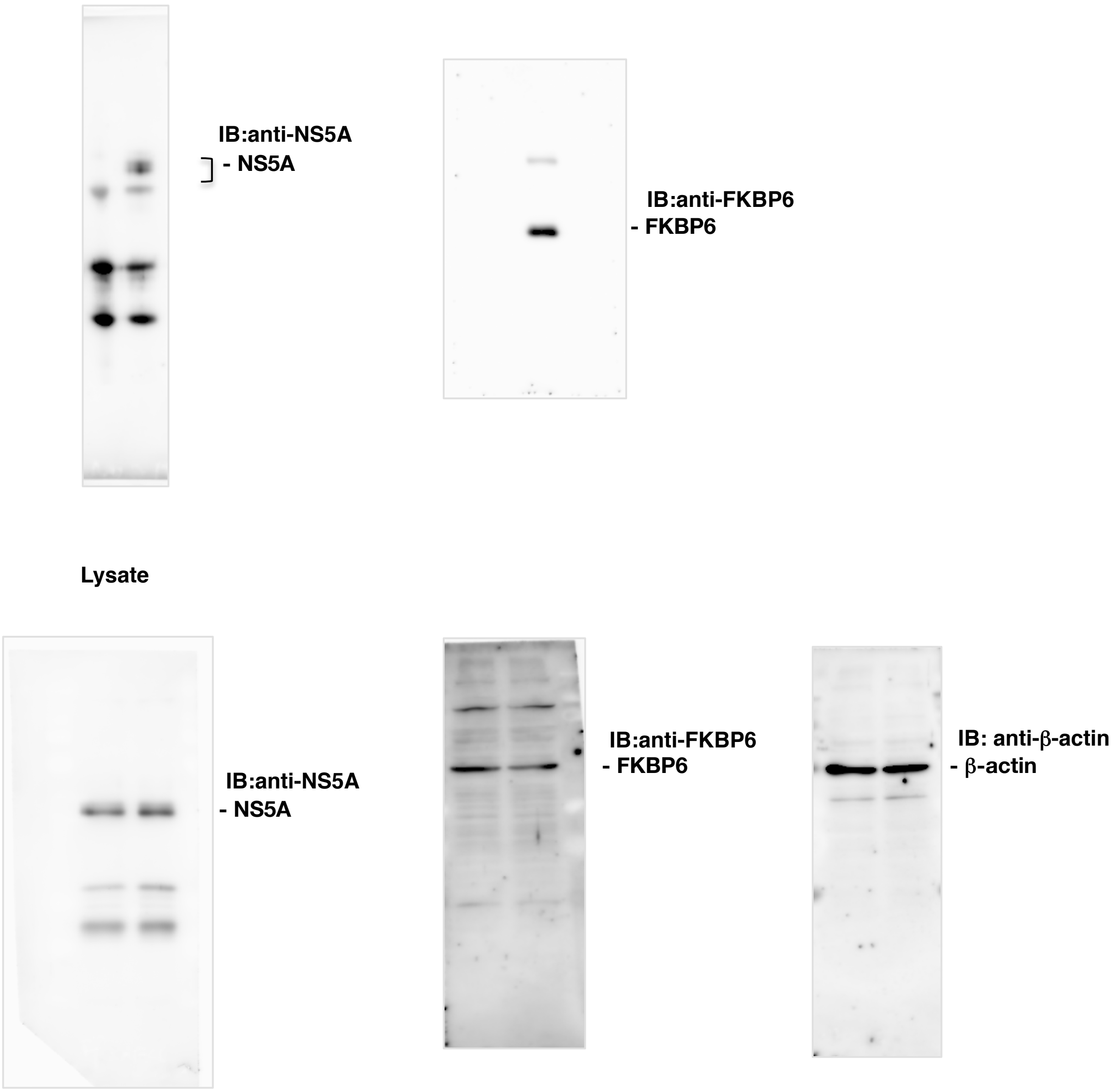

Supplementary Figure 3. Original data of Figure 1D.

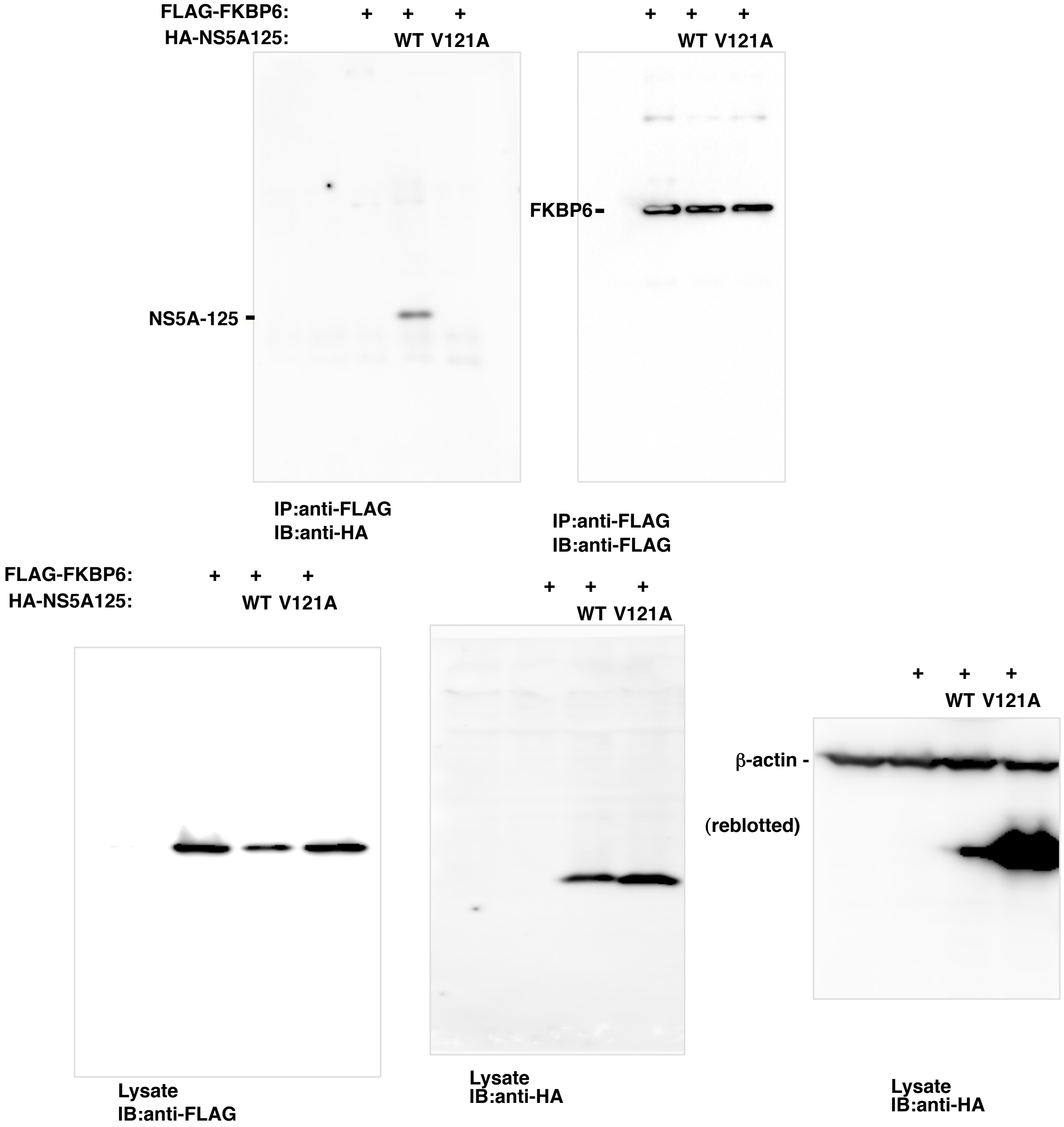

Supplementary Figure 4. Original data of Figure 3A.

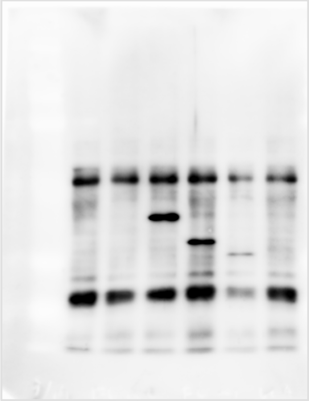

IP:anti-FLAG
IB:anti-HA

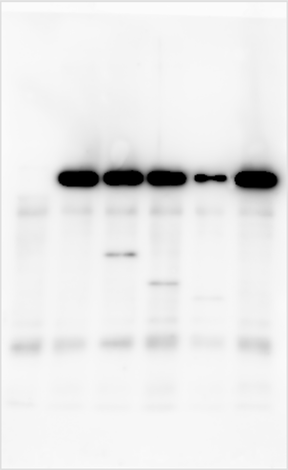

IP:anti-FLAG
IB:anti-FLAG

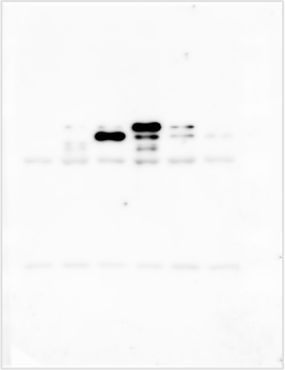

IP:anti-HA
IB:antiFLAG

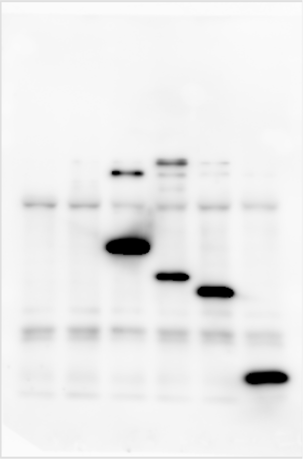

IP:anti-HA
IB:anti-HA

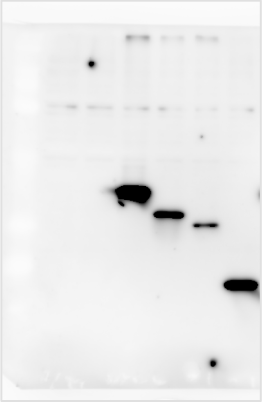

Lysate
IB:anti-HA

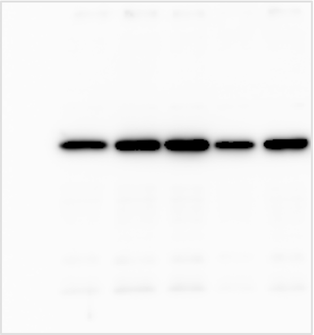

Lysate
IB:anti-FLAG

Lysate
IB: anti-b-actin

Supplementary Figure 5. Original data of Figure 3 C.

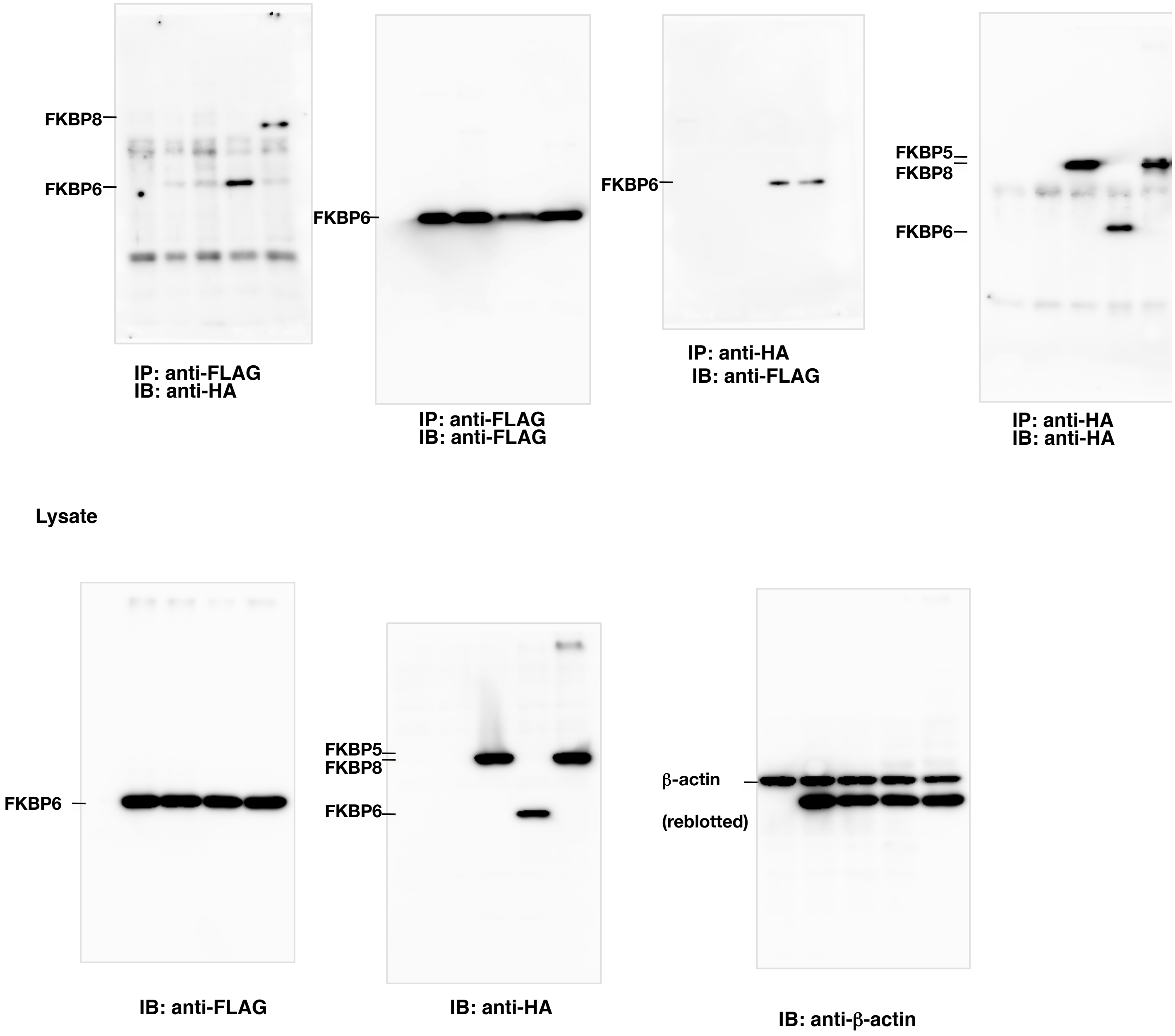

Supplementary Figure 6. Original data of Figure 4A.

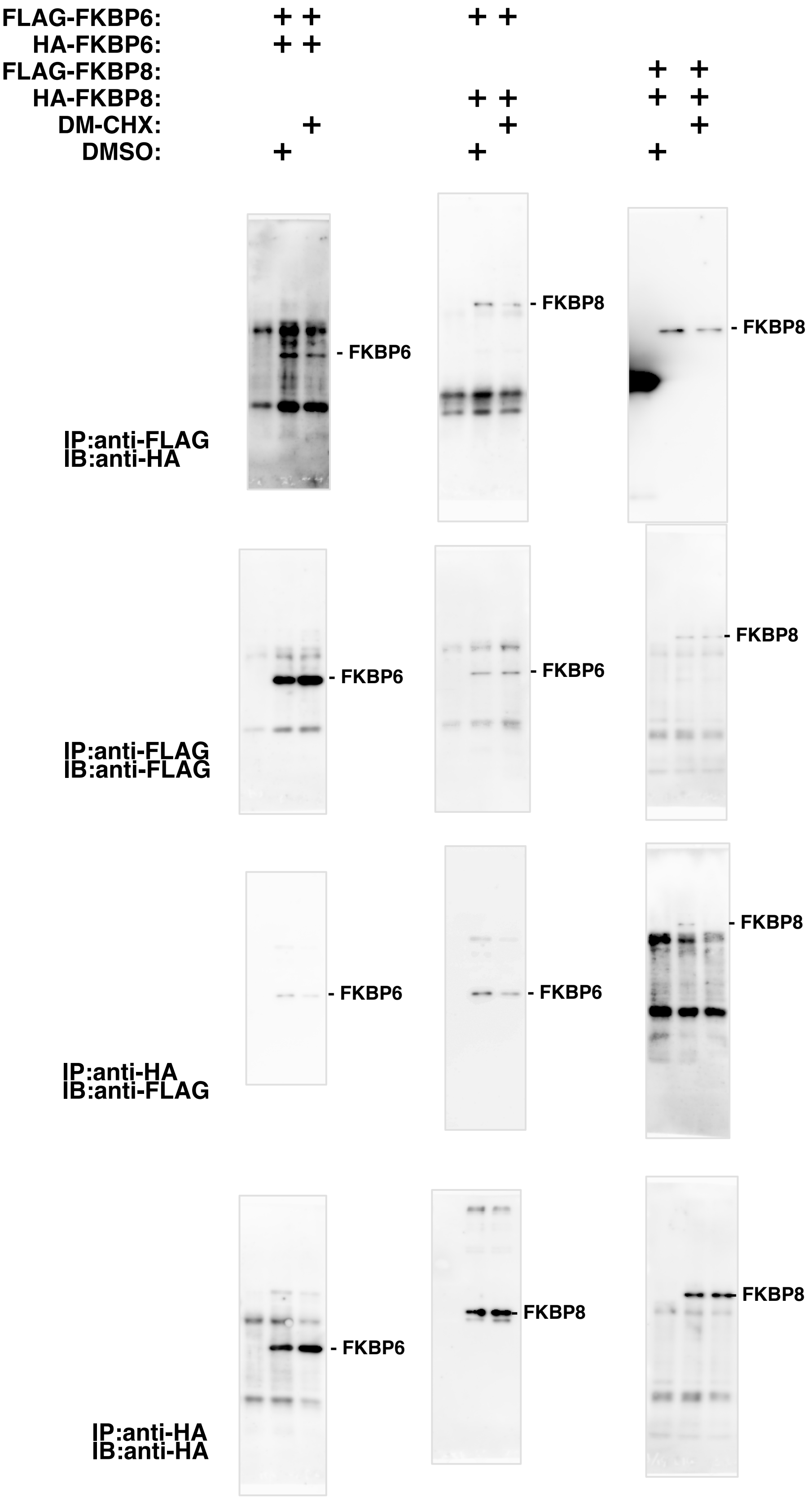

Supplementary Figure 7. Original data of Figure 4D.

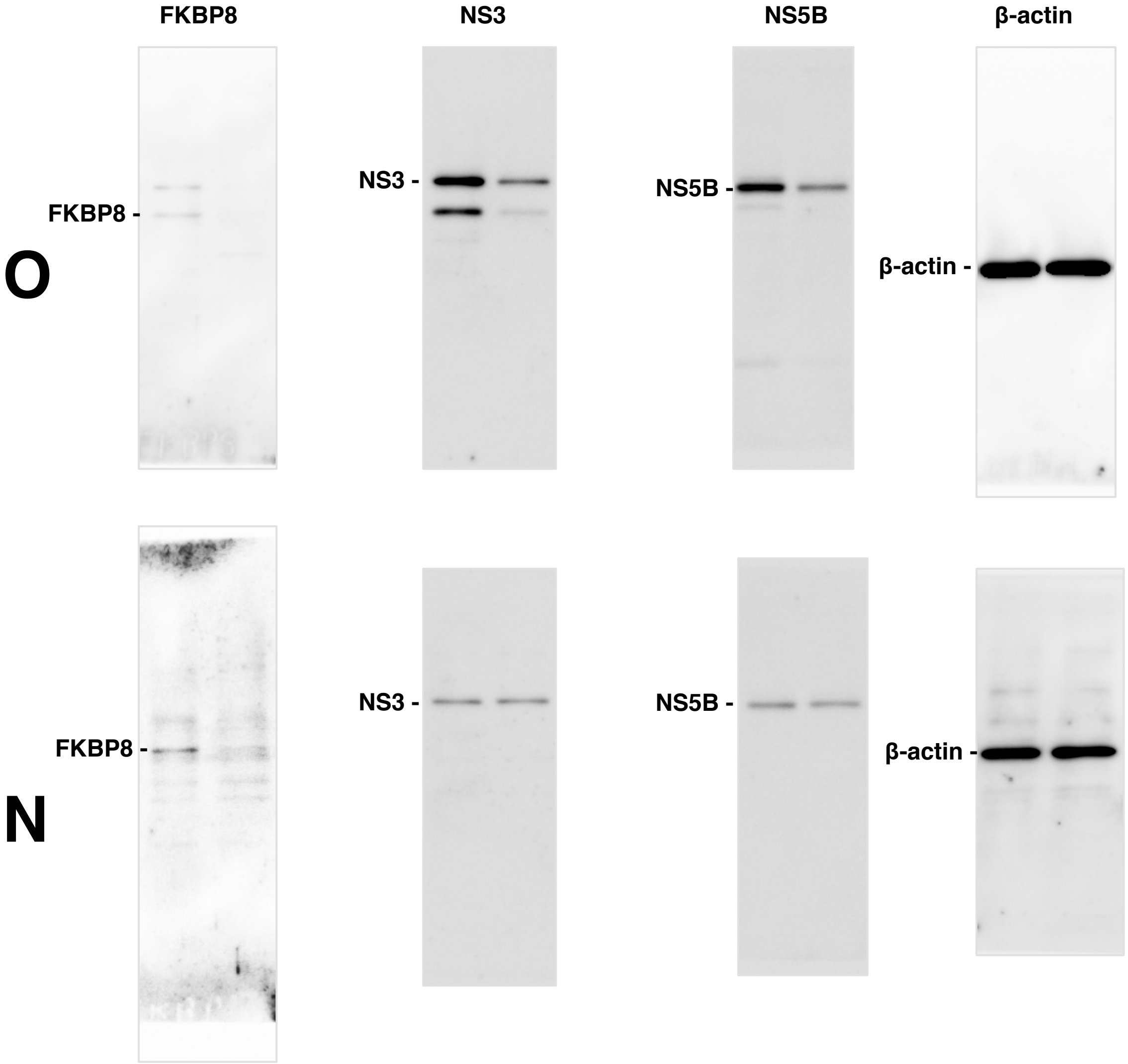

Supplementary Figure 8. Original data of Figure 5B.

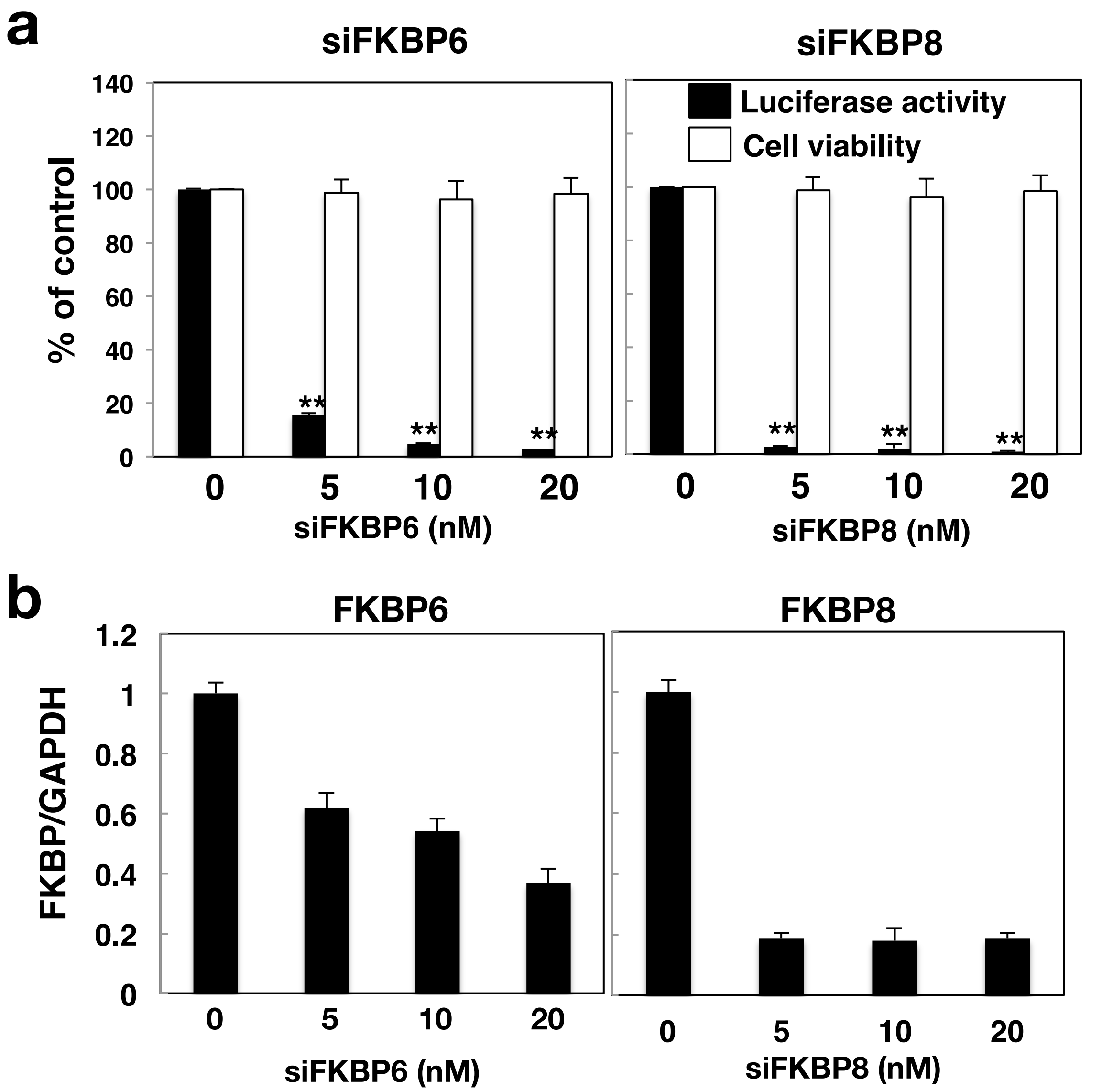

Supplementary Figure 9. Dose dependency of siRNA targeting FKBP6 or FKBP8 on HCV replication.

a: Effect of FKBP knockdown on HCV replication. Luciferase activity and cell viability were measured in the O replicon cell line transfected with various amounts of siFKBP6 or siFKBP8. The total amount of transfected siRNA was adjusted with siControl. Transfected cells were harvested at 72 h post-transfection in order to measure luciferase activity and cell viability. The values obtained were standardized with a value of 0 nM (control) and represented as percentages. Asterisks indicate a significant difference from the control value (**: $P < 0.01$). The data shown in this figure are representative of three independent experiments. **b:** The amounts of FKBP6 and FKBP8 in knockdwon cells. The amounts of mRNAs of FKBP6, FKBP8 and GAPDH were estimated by qRT-PCR.

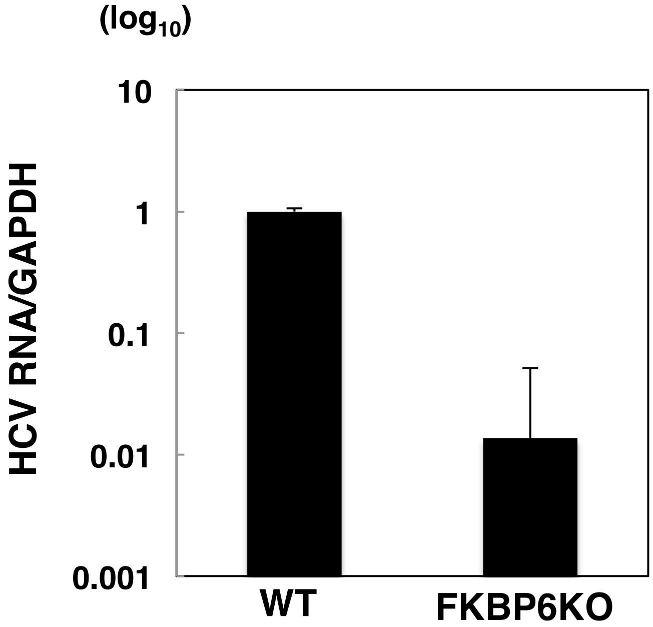

Supplementary Figure 10. Effect of FKBP6 knockout on HCV infection.

Huh7OK1 cells (WT) and FKBP6-knockout Huh7OK1 cells (FKBP6KO) were infected with HCVcc at an m.o.i. of 0.5 and then harvested 4 days post-infection. The HCV RNA and GAPDH mRNA were estimated by qRT-PCR. The value of HCV RNA was normalized with the value of GAPDH mRNA. Asterisks indicate a significant difference of a pair (*: $P < 0.05$). The data shown in this figure are representative of three independent experiments.

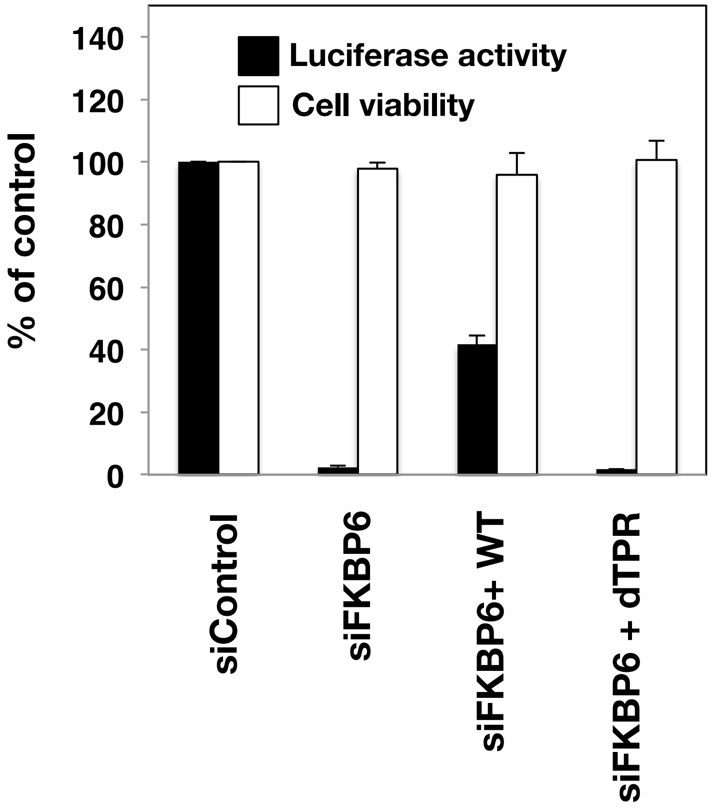

Supplementary Figure 11. Expression of FKBP6 lacking TPR domains did not recover HCV replication in FKBP6-knockdown replicon cells.

HCV replicon cell lines were transfected with siFKBP6 or siControl at a final concentration of 10 nM. The resulting cells were incubated for 16 hours and then transfected with 1 μg of the plasmid encoding HA-FKBP6 (WT) or HA-dTPR3 (dTPR). The transfected cells were incubated for 56 h and then harvested in order to estimate luciferase activity and cell viability.

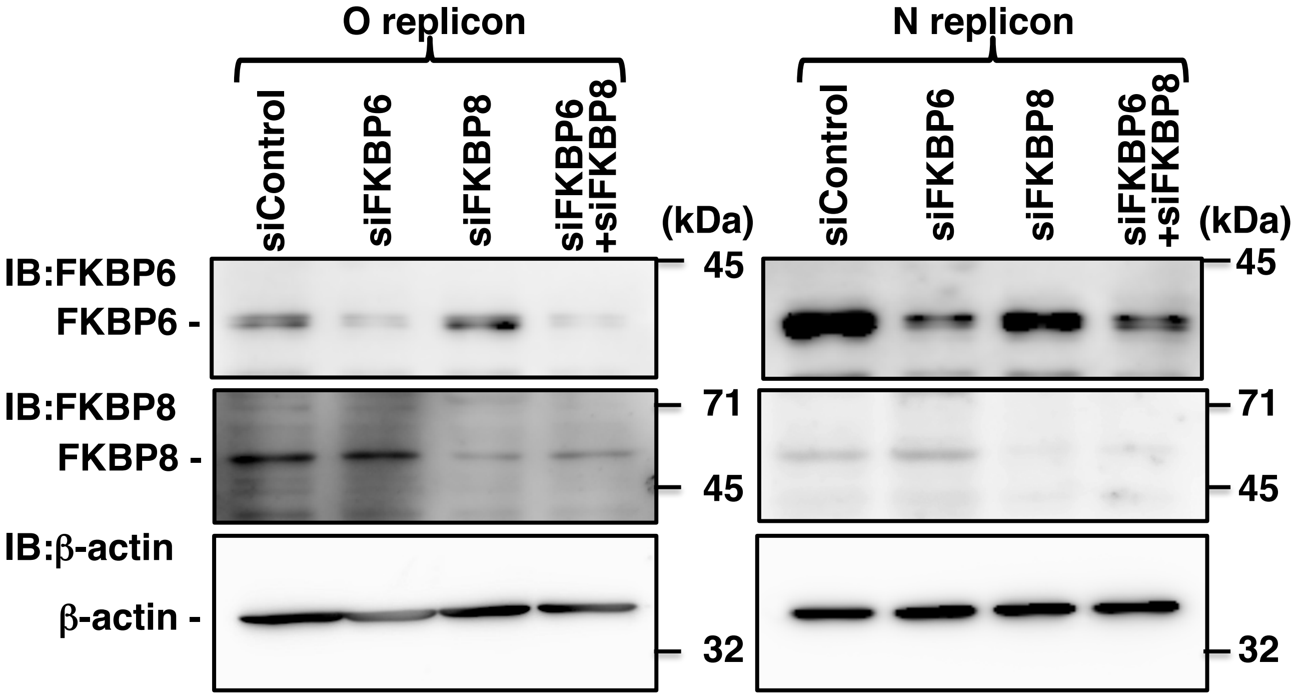

Supplementary Figure 12. Immunoblotting analysis of FKBP6 and FKBP8 protein in knockdown cells. Immunoblotting data were shown using samples of Figure 5d.

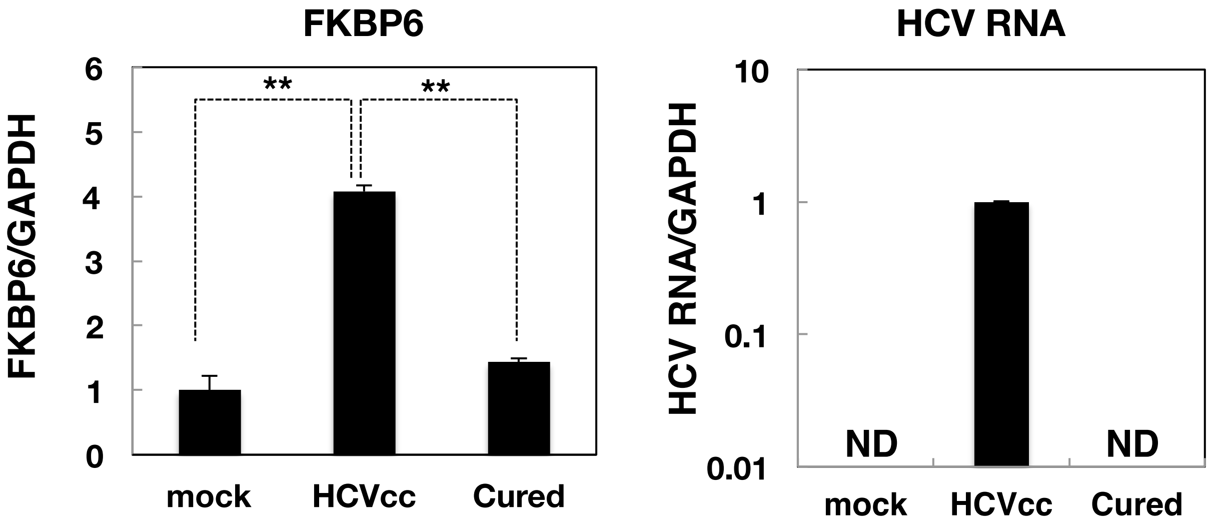

upplementary Figure 13. Effects of HCV elimination on FKBP6 expression in HCV infected cells.

HuhOK1 cells persistently infected with HCV were treated with 1 μM daclatasvir for 3 weeks in order to eliminate HCV. HCV RNA and FKBP6, FKBP8, and GAPDH mRNAs in naïve (mock), HCVcc-infected cells (HCVcc) and cured cells (Cured) were estimated by qRT-PCR. Asterisks indicate a significant difference from the value of "HCVcc" (**: $P < 0.01$). ND means "not detected".

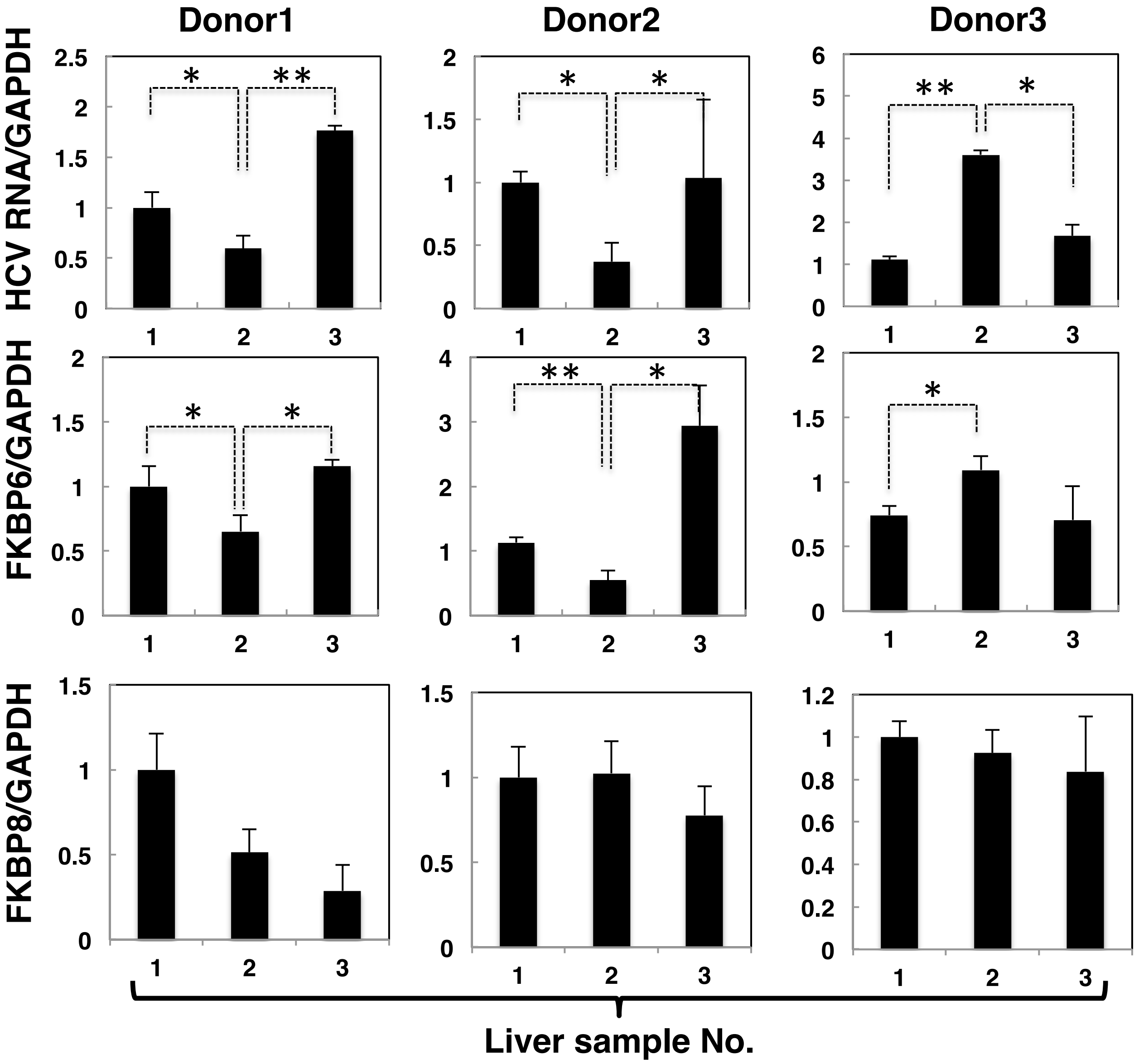

Supplementary Figure 14. Effect of HCV infection on expression of FKBP6. Human non-cancerous liver tissues were collected from three independent donors (Donor 1, 2 and 3). Three aliquots (no. 1, 2 and 3) were cut from different corresponding positions of a liver sample of each donor. HCV RNA and mRNAs of FKBP6, FKBP8, and GAPDH were estimated by qRT-PCR. The values obtained were normalized with GAPDH mRNA. Asterisks indicate a significant difference of a pair (*: $P < 0.05$, **: $P < 0.01$). The data shown in this figure are representative of three independent experiments.
